# Agonistic anti-DCIR antibody inhibits ITAM-mediated inflammatory signaling and promotes immune resolution

**DOI:** 10.1172/jci.insight.176064

**Published:** 2024-05-23

**Authors:** Liang Chen, Suresh Patil, Jeffrey Barbon, James Waire, Stephen Laroux, Donna McCarthy, Mishra Pratibha, Suju Zhong, Feng Dong, Karin Orsi, Gunarso Nguyen, Yingli Yang, Nancy Crosbie, Eric Dominguez, Arun Deora, Geertruida Veldman, Susan Westmoreland, Liang Jin, Timothy Radstake, Kevin White, Hsi-Ju Wei

**Affiliations:** 1 AbbVie, Cambridge Research Center, Cambridge, Massachusetts, USA.; 2 AbbVie Bioresearch Center, Worcester, Massachusetts, USA.; 3 AbbVie Bay Area, South San Francisco, California, USA.

**Keywords:** Autoimmunity, Immunology, Autoimmune diseases, Cellular immune response, Innate immunity

## Abstract

DC inhibitory receptor (DCIR) is a C-type lectin receptor selectively expressed on myeloid cells, including monocytes, macrophages, DCs, and neutrophils. Its role in immune regulation has been implicated in murine models and human genome-wide association studies, suggesting defective DCIR function associates with increased susceptibility to autoimmune diseases such as rheumatoid arthritis, lupus, and Sjögren’s syndrome. However, little is known about the mechanisms underlying DCIR activation to dampen inflammation. Here, we developed anti-DCIR agonistic antibodies that promote phosphorylation on DCIR’s immunoreceptor tyrosine-based inhibitory motifs and recruitment of SH2 containing protein tyrosine phosphatase-2 for reducing inflammation. We also explored the inflammation resolution by depleting DCIR^+^ cells with antibodies. Utilizing a human DCIR–knock-in mouse model, we validated the antiinflammatory properties of the agonistic anti-DCIR antibody in experimental peritonitis and colitis. These findings provide critical evidence for targeting DCIR to develop transformative therapies for inflammatory diseases.

## Abstract 2

Agonistic anti-DCIR antibodies reduce inflammation by inhibiting proinflammatory cytokine production and suppressing leukocyte accumulation, and have potential for treating inflammatory diseases.

## Introduction

Immune regulator receptors play a crucial role in immune homeostasis by balancing activating versus inhibitory immune responses. The inhibitory receptors usually transduce the inhibitory signal through immunoreceptor tyrosine-based inhibitory motifs (ITIMs) located in the intracellular domains to counteract the functions of immunoreceptor tyrosine-based activation motif–bearing (ITAM-bearing) receptors in a proximity. This fine-tuned regulation from 2 opposed signaling modules can be initiated by receptors interacting with antibodies, immune complexes, or opsonized particles in a multivalency manner to control the ultimate cellular responses. The insufficient inhibitory signals by the dysfunctional ITIM-bearing receptors can affect the immune responses and eventually lead to autoimmune disorders (1, 2).

DC inhibitory receptor (DCIR) belongs to the C-type lectin receptor (CLR) family and is predominantly expressed on myeloid-derived cells including monocytes, DCs, macrophages, and neutrophils. DCIR contains an extracellular lectin–like domain and an ITIM motif in the cytoplasmic domain (3) to provide antiinflammatory signaling through the recruitment of phosphatases SHP-1 and SHP-2 (4, 5). DCIR^–/–^ mice are highly susceptible to disease onset in collagen-induced arthritis (CIA) (6) and experimental autoimmune encephalomyelitis (EAE) models (7). Furthermore, DCIR polymorphisms are linked to patients’ susceptibility to autoimmune diseases such as rheumatoid arthritis (RA) (8), systemic lupus erythematosus (SLE) (9), and primary Sjögren’s syndrome (9). Therefore, insufficient DCIR function might cause autoimmunity.

Studies have demonstrated that DCIR receptor in DCs and macrophages inhibits production of TLR-dependent inflammatory cytokines, such as IL-1β, IL-6, TNF-α, IL-12, and IFN-α (10–12). Ligation of DCIR by sialylated IgG generates tolerogenic DCs and Tregs (4). A recent study demonstrated that an asialo-biantennary N-glycan(s) (NA2) binding to DCIR on DCs ameliorate CIA and EAE symptoms (13). These studies indicate that engagement of DCIR with ligands mediates the resolution of inflammation. However, the mechanisms that govern this inhibitory receptor biology by agonism are not well understood. In addition, an optimized agonistic antibody needs to be developed to induce potent and selective myeloid cell inhibition via targeting DCIR.

Here, we generated a panel of antihuman DCIR (anti-huDCIR) mAbs and identified 2 strongly agonistic mAbs (clones 3A4 and 9D9). We found that the agonistic anti-DCIR mAbs induced ITIM phosphorylation and SHP2 recruitment to DCIR in human monocytes and DCs, diminished the Syk-SHP2-ITAM association triggered by the ITAM-bearing receptors activation, and reduced the proinflammatory cytokine production in both human monocytes and peripheral blood mononuclear cells (PBMCs). Additionally, in huDCIR–knock-in (huDCIR-KI) mouse peritonitis and colitis models, there was more DCIR^+^ cell accumulation in the peritoneal fluid and colonic crypts, respectively, compared with naive mice. Importantly, treatment of agonistic but not nonagonistic mAbs significantly reduced the DCIR^+^ cell accumulation in the peritonitis and colitis models. Moreover, 1 weak-agonistic clone, 5E11 with an engineered mouse IgG2b Fc to enhance the antibody-dependent cell depletion also suppressed leukocyte/neutrophil accumulation and the proinflammatory cytokine production in the peritonitis model. Taken together, these findings reveal the immune inhibitory mechanisms through agonistic and cell depletion effects provided by anti-DCIR mAbs and lay a solid foundation for targeting DCIR in the pursuit of transformative therapies for inflammatory diseases.

## Results

### DCIR expression is induced in the disease-associated myeloid antigen-presenting cells and neutrophils.

**Figure 1:**
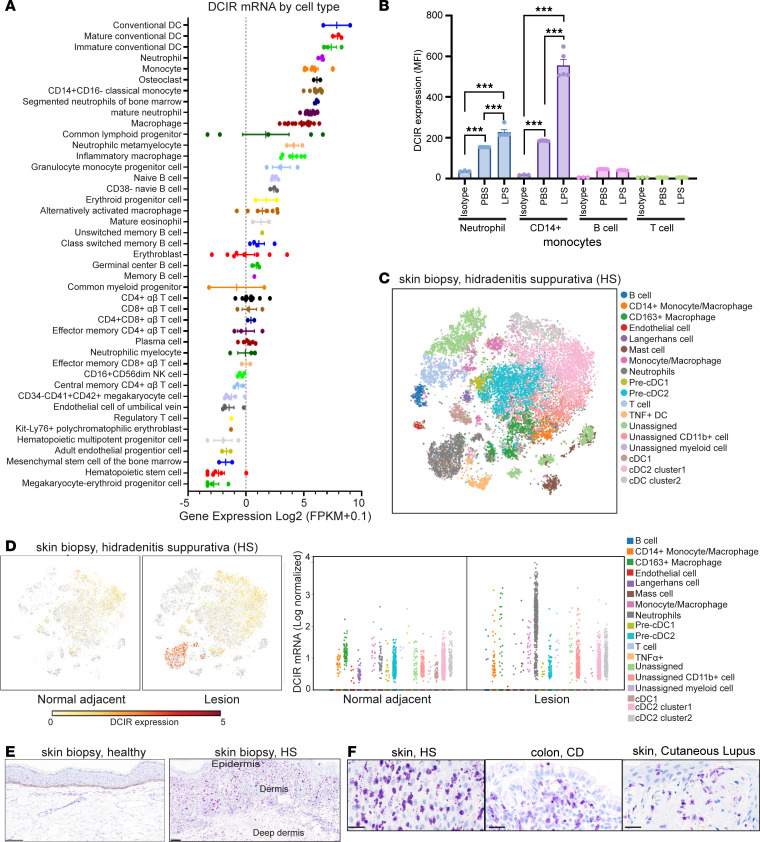
DCIR expression is induced in the disease-associated antigen-presenting myeloid cells and neutrophils. (**A**) Relative DCIR mRNA expression in blood immune cells (BLUEPRINT data set). (**B**) Flow cytometry analysis of DCIR expressed in the neutrophils, monocytes, and T and B cells isolated from human PBMCs with or without 100 ng/mL LPS stimulation for 1 hour (*n* = 3 for isotype staining control, *n* = 5 for PBS or LPS-treated group). Means ± SEM are shown, and statistical analysis is determined by 1-way ANOVA test with Dunnett’s correction compared with the isotype staining control. ***P < 0.001. (**C**) t-SNE plot of single-cell RNA-Seq analysis for skin biopsy collected from hidradenitis suppurativa (HS) patients (GSE155850). (**D**) DCIR-expressing cells overlaying on t-SNE plot of the single-cell RNA-Seq analysis for the normal adjacent tissue or skin lesion from patients with HS. DCIR mRNA level in the different cell clusters were quantitated by pseudo-bulk differential expression analysis based on scRNA-Seq results as described in **C**. (**E**) Representative immunohistochemical staining of DCIR^+^ cells in normal or disease skin tissues of HS. (**F**) Immunohistochemical staining of DCIR^+^ cells in skin lesion of HS and SLE, and mucosal tissue of CD. Scale bars: 100 μm.

To confirm DCIR expression, we first compared DCIR mRNA expression in unperturbed blood immune cells based on the BLUEPRINT data set (Figure 1A). DCIR expression is enriched in not only immature and mature conventional DCs (cDCs) but also on neutrophils, classical monocytes, and macrophages. However, DCIR expression is low on B cells and T cells. Like other immune checkpoint receptors, elevated DCIR after stimulation can be a feedback mechanism to prevent excessive inflammation in activated immune cells (14). Indeed, an RNA-Seq metastudy shows elevated levels of DCIR in patients with autoimmune diseases, including Crohn’s disease (CD), ulcerative colitis (UC), and hidradenitis suppurativa (HS), compared with healthy samples (Supplemental Figure 1A; supplemental material available online with this article; https://doi.org/10.1172/jci.insight.176064DS1). Consistent with the mRNA profiling results, we verified DCIR protein expressed on the surface of neutrophils and monocytes from human whole blood by FACS (Supplemental Figure 1B), which was further induced in the inflammatory condition due to LPS stimulation (Figure 1B). Little DCIR was identified on the B and T cells, even when stimulated with LPS (Figure 1B).

To scrutinize the cells contributing to the increased DCIR in the disease-associated tissues, we analyzed public single-cell RNA-Seq (scRNA-Seq) data sets derived from the diseased skin tissues of patients with HS (GSE155850) (Figure 1, C and D) and mucosal tissues of patients with CD (GSE134809) (Supplemental Figure 1, C and D). The scRNA-Seq analysis revealed that DCIR is enriched in the tissue-infiltrating neutrophils, monocytes, macrophages, and tissue-resident myeloid cells including cDC1/2 and Langerhans cells in the lesional HS samples (Figure 1D). Beyond HS, mucosal tissue scRNA-Seq data (GSE134809) from patients with CD with resistance to anti–TNF-α therapy revealed that DCIR is selectively expressed on classical and intermediate monocytes and on DCs in the lesions (BioTuring software v2.0.5; Supplemental Figure 1, C–E). Mucosal tissue bulk RNA-Seq data further support that enhanced DCIR expression is associated with antiinflammatory treatment resistance (Supplemental Figure 1F), as patients who responded to anti–TNF-α therapy displayed significantly lower DCIR levels before treatment compared with the treatment nonresponders. In agreement with RNA-Seq results, immunohistology staining also confirmed increased DCIR^+^ cell accumulation in both epidermis and dermis of the HS skin biopsy, CD mucosal tissue, and a cutaneous lupus skin lesion (Figure 1, E and F). The highly increased DCIR expression in disease lesions provides the rationale for developing DCIR-based therapy to restrain the inflammatory myeloid cell migration and activation.

### Characterization of humanized anti-DCIR mAbs.

To investigate the potential of DCIR as a therapeutic target for treating inflammatory diseases, we initiated an antibody generation campaign to produce anti-DCIR mAbs (Supplemental Figure 2A). Briefly, we immunized rats with a huDCIR cDNA vector and generated hybridoma cells by fusing the resulting spleen B cells with myeloma cells. We screened the culture supernatants of isolated hybridoma clones for secreted antibodies that bound to huDCIR-overexpressing HEK293 cells and monocyte-derived DCs (MoDCs) (Supplemental Figure 2B). The heavy and light chain variable regions (VH and VL) of the hybridomas that secreted strong DCIR binders were cloned into a human IgG1 backbone and expressed as chimeric mAbs. Their cross-reactivity to cynomolgus (cyno) DCIR and huDCIR, but not murine DCIR1 (muDCIR1) were also confirmed (Supplemental Figure 2C). Finally, fully humanized antibodies were produced after removing rat-derived sequences and codon-optimization suggested by the high-throughput antibody humanization design software developed at AbbVie. The resultant humanized antibodies that passed quality controls for aggregation and thermal stability (data not shown) were confirmed to be specific to human and cyno DCIR but not other selected human CLRs, pattern recognition receptors, or muDCIR1 expressed on immortal murine DC cell line JAWS II (Supplemental Figure 2D).

### Agonistic anti-DCIR mAb induces tyrosine phosphorylation and SHP2 recruitment to DCIR ITIM motif.

**Figure 2:**
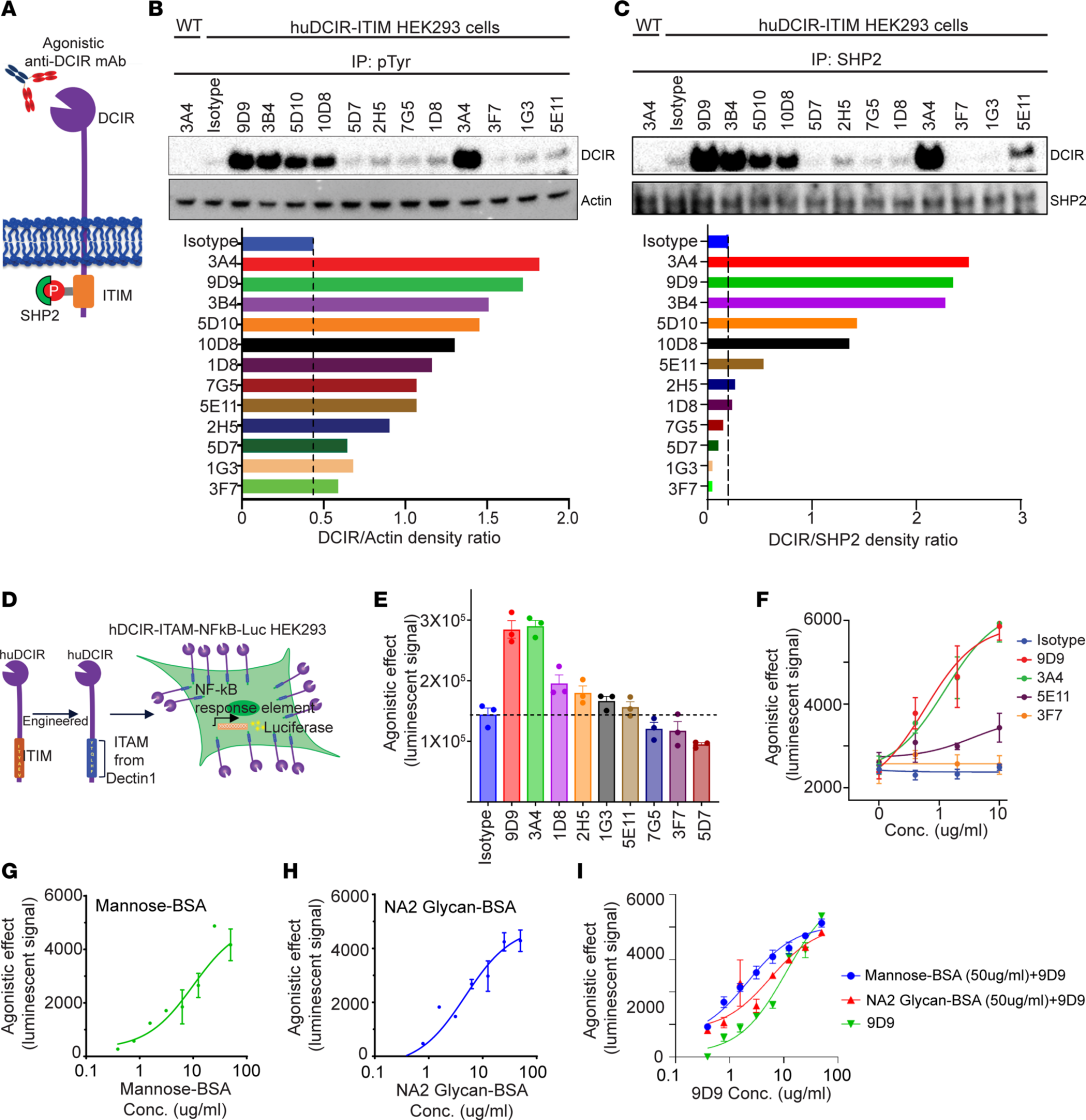
Agonistic anti-DCIR mAb induces tyrosine phosphorylation and SHP2 recruitment to DCIR ITIM motif. (**A**) Graphic of the agonistic effect induced by anti-DCIR mAbs. (**B** and **C**) WT and huDCIR transfected HEK293 cells were treated with anti-DCIR mAbs (10 μg/mL) for 30 minutes, followed by immunoprecipitation assay (IP) with anti-phosphorylated tyrosine (**B**) or anti-SHP2 (**C**) antibodies. DCIR levels were analyzed by WB and quantitated by densitometry. Representative data from 2 independent studies are shown. (**D**) Illustration of the huDCIR agonistic effect reporter cell generation. HEK293 cell was cotransfected with a huDCIR vector containing an ITAM motif cloned from Dectin-1 and a luciferase reporter vector with NF-кB response element. (**E**) Reporter cells as described in **D** were treated with 10 μg/mL anti-DCIR mAbs or isotype control for 30 minutes, followed by luciferase assay analysis. Agonistic effects induced by the antibodies were quantitated by the luminescent signal. (**F**) Reporter cells as described in **D** were treated with a serial dilution of agonistic or nonagonistic anti-DCIR antibodies or isotype control for 30 minutes, followed by luciferase assay analysis. Dose-dependent induction of agonistic signaling was quantitated by the luminescent signal. (**G** and **H**) DCIR ligands, mannose and asialo-biantennary N-glycan (NA2-glycan), were conjugated with BSA and immobilized on culture plates. Dose-dependent agonistic effects of mannose-BSA and NA2 glycan-BSA were determined by the reporter system as described in **D**. (**I**) In total, 50 μg/mL of mannose-BSA and NA2 glycan-BSA were coated on the cell culture plate and cultured with HEK293 cells contain the huDCIR-agonist reporter for 6 hours. Agonistic effects induced by anti-DCIR antibody (9D9) were determined by luciferase assay after overnight culture. (**E**–**I**) Representative data from 2 independent studies are shown. Means ± SEM from triplicates are shown.

Although the suppressive role of DCIR has been reported in multiple inflammatory disease models (6, 7, 15), its intracellular signaling remains unclear. Previous studies have shown that the ITIM motif of checkpoint receptors PD-1, SIGLEC9, and SIRPα can be phosphorylated at tyrosine residues upon agonistic ligand binding, which then recruits SHP2 (16–19). DCIR association with SHP2 was also observed in the DCIR-overexpressed BM-derived DCs (BMDCs) (20). Based on these findings, we hypothesize that agonistic anti-DCIR mAbs could trigger tyrosine phosphorylation and promote SHP2 recruitment to DCIR (Figure 2A). To evaluate this hypothesis, we screened anti-DCIR mAbs in huDCIR-overexpressing HEK293 cells based on tyrosine phosphorylation and SHP2 interaction of DCIR identified by immunoprecipitation. We identified 3 mAbs — clones 9D9, 3B4, and 3A4 — that induced strong agonistic effects (Figure 2, B and C).

To enable a high-throughput comparison of agonistic signaling triggered by in-house–generated humanized anti-DCIR mAbs, we created a DCIR agonistic signaling reporter cell line by cotransfecting HEK293 cells with an NF-κB luciferase reporter vector and a chimera DCIR vector carrying the intracellular ITAM domain from Dectin-1 to replace the DCIR ITIM motif (Figure 2D). We quantified antibody-mediated agonistic effects by measuring luciferase activity through ITAM/NF-κB signaling activation. Using this system, we selected 2 strong agonistic anti-DCIR mAbs, 9D9 and 3A4, as well as a non- and a weak-agonistic anti-DCIR mAbs, 3F7 and 5E11, for scale-up production and downstream mechanistic studies (Figure 2, E and F). Mannose and NA2-glycan have been reported as DCIR’s ligand for inducing DC tolerance (13, 21). We confirmed that mannose and NA2-glycan conjugated with BSA and immobilized on plates can induce an agonistic effect, which was saturated at 50 μg/mL using the DCIR agonistic signaling reporter system (Figure 2, G and H). However, pretreatment of mannose-BSA or NA2-glygan-BSA (50 μg/mL), followed by the addition of agonistic anti-DCIR antibody (9D9), did not compromise the signaling induced by 9D9 (Figure 2I), indicating that our agonistic anti-DCIR antibody further amplified immune tolerance signaling from DCIR even when occupied by natural ligands.

### Agonistic anti-DCIR mAb provides immunosuppressive function by sequestering SHP2 away from ITAM receptors.

**Figure 3:**
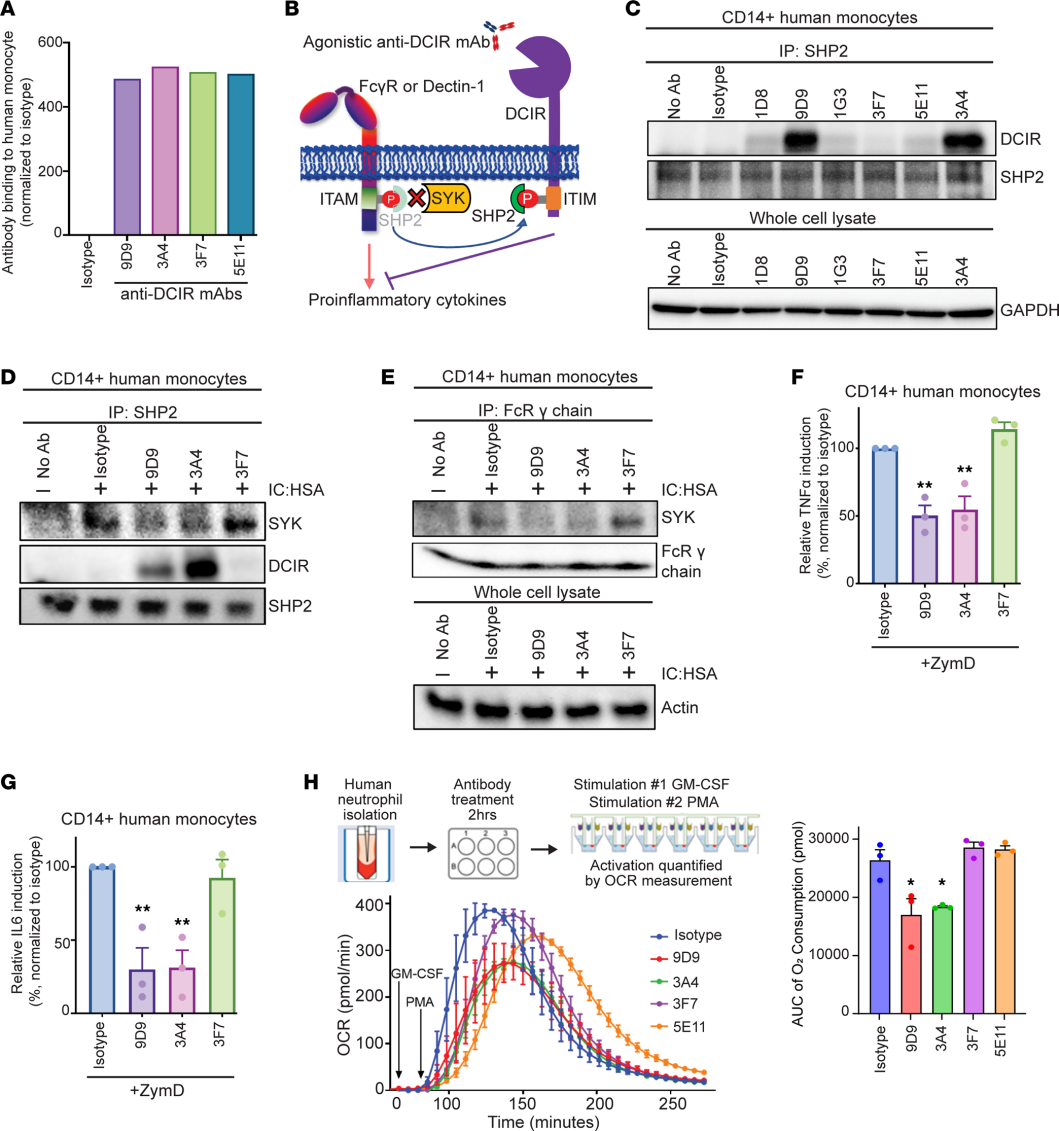
Agonistic anti-DCIR mAb provides immunosuppressive function by increasing SHP2 binding to the ITIM. (**A**) Human monocytes were treated with 5 μg/mL antibodies for 30 minutes. Antibody binding was quantitated by the 10 μg/mL PE anti-human IgG secondary antibody. Data are normalized to the isotype group. Representative data from 2 independent studies are shown. (**B**) Scheme of the immunosuppressive effect induced by the agonistic anti-DCIR mAb. (**C**) Human monocytes were treated with 5 μg/mL anti-DCIR mAbs or isotype for 30 minutes, followed by immunoprecipitation (IP) using anti-SHP2 antibody. SHP2-DCIR interaction was evaluated by the DCIR level analyzed by WB. SHP2 from IP lysate and GAPDH from whole cell lysate were probed as loading controls. (**D** and **E**) Human monocytes were pretreated with 5 μg/mL antibodies for 30 minutes, followed by 50 μg/mL anti-human IC: HSA stimulation for 30 minutes. SYK’s interactions with SHP2 and FcRγ chain were evaluated by immunoprecipitation using anti-SHP2 (**D**) and anti-FcRγ (**E**) antibodies. SHP2 and FcRγ chain from IP lysate and Actin from whole cell lysate were probed as loading controls. (**A** and **C**–**E**) Representative data from 2 studies are shown. (**F** and **G**) Human monocytes (*n* = 3) were pretreated with 10 μg/mL antibodies for 30 minutes, followed by 25 μg/mL ZymD stimulation overnight. Induction of TNF-α and IL-6 in supernatant were measured by ELISA and normalized to the isotype group. (**H**) Human neutrophils were pretreated with 10 μg/mL antibodies for 2 hours, and OCR were detected in real time after GM-CSF/PMA stimulation. AUC of the OCR was shown. Representative data from 3 independent studies are shown. (**F**–**H**) Means ± SEM are shown, and statistical significance is determined by 1-way ANOVA test with Dunnett’s correction compared with the isotype condition. *P < 0.05, **P < 0.01.

After selecting the lead humanized agonistic anti-DCIR mAbs, we investigated how DCIR agonistic signaling influences human primary cell activation. To ensure that the agonistic effect was not due to antibodies’ binding discrepancy, we confirmed their comparable binding to human monocytes (Figure 3A). SHP2 has been reported to regulate inflammatory signaling in a dual manner. On one hand, SHP2 functions as a phosphatase and negatively modulates inflammatory signaling (22–24). On the other hand, SHP2 can act as an adaptor protein and recruit SYK to ITAM-containing receptors, such as Dectin-1 and Fc receptor γ (FcRγ) chain, promoting cell activation (25). Therefore, we hypothesize that DCIR’s ITIM can compete with ITAM for SHP2, and SHP2 binding to DCIR prevents SYK activation and interaction with the ITAM-bearing receptor (Figure 3B).

Consistent with the antibody screening results using DCIR-overexpressing HEK293 cells, agonistic anti-DCIR mAbs 9D9 and 3A4 triggered DCIR and SHP2 interaction in human monocytes (Figure 3C). Stimulating the monocytes with antihuman serum albumin immune complex (IC: HSA) in the presence of Fc-matched isotype control or nonagonistic anti-DCIR mAb 3F7 increased the interaction between SYK and SHP2 (Figure 3D). However, treating the immune complex–stimulated monocytes with agonistic anti-DCIR mAbs 9D9 and 3A4 significantly reduced the SHP2-SYK interaction to a similar level observed in cells with no stimulation, while promoting the SHP2 and DCIR interaction (Figure 3D). These data suggest that DCIR/ITIM signaling might overcome ITAM activation and compete for SHP2 recruitment. Indeed, agonistic anti-DCIR clones 9D9 and 3A4 also compromised the interaction between SYK and FcRγ chain (Figure 3E). Another ITAM receptor, DECTIN1, also requires the formation of an ITAM-SHP2-SYK signaling complex (25). We found that agonistic mAbs 9D9 and 3A4 suppressed DECTIN1 activation in response to zymosan-D (ZymD) stimulation, as indicated by the reduced TNF-α and IL-6 secretion from monocytes (Figure 3, F and G) and PBMC (Supplemental Figure 3, A–D), whereas the nonagonistic 3F7 showed no effect. Beyond pattern-recognition receptors, SHP2 is also required for the function of GM-CSF (26), regulating the differentiation and activation of neutrophils and macrophages. Given the rapid neutrophils’ activation and ROS production kinetics, we analyzed anti-DCIR antibodies’ effect on the oxidative burst of neutrophils stimulated with GM-CSF/phorbol myristate acetate (PMA) in real time by measuring OCR, which reflects the oxygen consumption by NOX2 to generate ROS (27). Both agonistic antibodies (9D9 and 3A4) reduced OCR compared with the isotype and non/weak-agonistic antibodies (3F7 and 5E11) (Figure 3H).

### Infiltrated DCIR^+^ cells are evident in huDCIR-KI mice during acute peritonitis and colitis.

**Figure 4:**
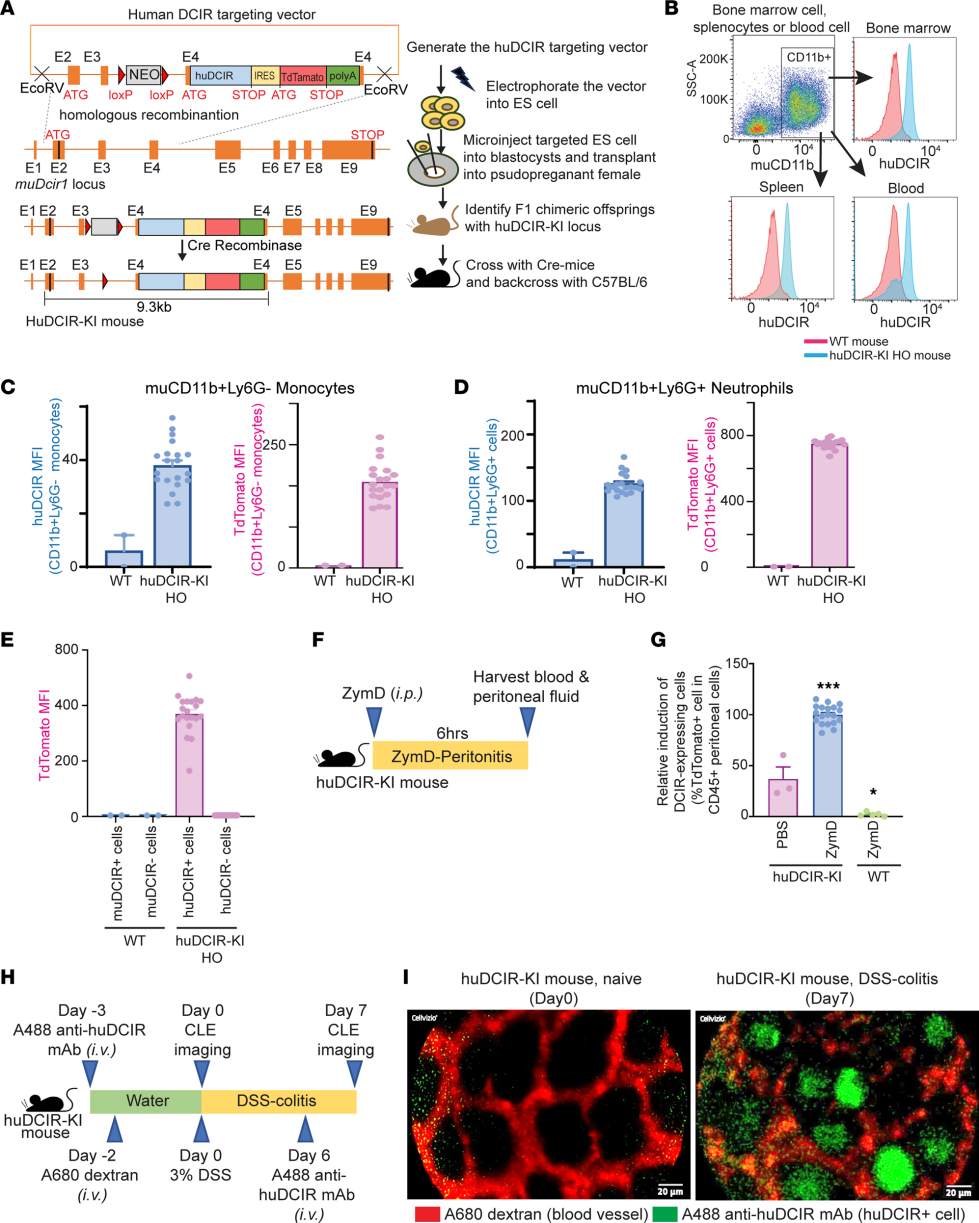
Infiltrated DCIR^+^ cells are evident in huDCIR-KI mice during the acute peritonitis and colitis models. (**A**) Illustration of the huDCIR-KI mice generation. (**B**) Flow cytometry of huDCIR expressed in pooled BM spleen, or blood CD11b^+^ cells from WT or homogenous huDCIR-KI mice (HO). (**C**–**E**) Flow cytometry of huDCIR and tdTomato expressed in the monocytes (CD11b^+^Ly6G^–^), neutrophils (CD1b^+^Ly6G^+^), and DCIR^+^ and DCIR^–^ cells from the BM of WT or homogenous huDCIR-KI mice. (**F**) Design of the peritonitis model induced by i.p. injection of 0.5 mL 2 mg/mL ZymD. (**G**) Flow cytometry quantitation of huDCIR-expressing (tdTomato^+^) cells in the CD45^+^ leukocytes isolated from the peritoneal lavage of WT and homogenous huDCIR-KI mice in the peritonitis model as described in **F**. Means ± SEM are shown, and statistical analysis is determined by 1-way ANOVA test with Dunnett’s correction compared with the PBS treated huDCIR-KI group. *P < 0.05, ***P < 0.001. (**H**) Design of the DSS-colitis model. Mice were fed 3% DSS in drinking water for 7 days. For the vessel staining, mice were i.v. administrated with 100 μL of 1 mg/mL 10 kDa AF680 dextran 2 days before feeding DSS water. For the DCIR^+^ cells detection, AF488 anti-DCIR mAb were i.v. injected into the mice 3 days before and 6 days after DSS feeding. CLE analyses were performed on day 0 (naive) and day 7 (colitis) during the DSS-feeding phase. (**I**) Representative CLE of naïve mice and mice with colitis as described in **F**. DCIR^+^ cells in the colonic crypts were stained with AF488 anti-DCIR mAb (green), and blood vessels running along the crypt wall were stained with AF680 dextran (red). Scale bars: 20 μm.

We then generated a huDCIR-KI mouse strain aiming to evaluate the potential of targeting huDCIR as a treatment for inflammatory diseases. To do so, we inserted huDCIR cDNA adjacent to an internal ribosome entry site–tdTomato (IRES-tdTomato) cassette into the *muDcir1* (*Clec4a2*) exon4 (Figure 4A). We confirmed successful cloning of huDCIR in the heterozygous (HET) huDCIR-KI mice by Southern blot analysis (Supplemental Figure 4, A and B) of genomic DNA isolated from mice tail tips. We observed similar huDCIR expression compared with the endogenous WT muDCIR1 in CD115^–^Ly6G^+^ BM neutrophils isolated from HET mice (Supplemental Figure 4C), indicating that the promoter activity and translational efficiency between the huDCIR and endogenous muDCIR1 are at a comparable level. Subsequently, gene expression of *huDCIR*, *muDcir1*, and *tdTomato* reporter in blood monocytes from HET huDCIR-KI mice were also validated by quantitative PCR (qPCR) analysis compared with the WT mice and human monocytes (Supplemental Figure 4D). Before inbreeding the HET huDCIR-KI mice to obtain homozygous (HO) huDCIR-KI mice, huDCIR-KI mice were backcrossed to the WT C57BL/6 mice for 7 generations to minimize the chance of nontarget genetic modification. The resultant HO huDCIR-KI mice show stable endogenous huDCIR expression on CD11b^+^ cells purified from BM, spleen, and blood compared with the WT mice (Figure 4B). BM CD11b^+^Ly6G^–^ monocytes and CD11b^+^Ly6G^+^ neutrophils from huDCIR-KI mice displayed stable expression of huDCIR and tdTomato compared with WT mice (Figure 4, C and D).

In addition, both huDCIR^+^ cells frequency and tdTomato expression were higher from HO compared with HET huDCIR-KI mice (Supplemental Figure 4E). TdTomato^+^ cells from both HO and HET huDCIR-KI mice express huDCIR (Supplemental Figure 4F), and tdTomato is solely expressed by huDCIR^+^ cells but not huDCIR^–^ cells in HO huDCIR-KI mice (Figure 4E). Furthermore, we confirmed that the intracellular signaling of the transgenic huDCIR is intact when binding to an agonistic anti-huDCIR antibody, clone 3A4, indicated by an increased interaction between huDCIR and mouse endogenous SHP2 in the BMDCs from HO huDCIR-KI mice (Supplemental Figure 4G). These results confirm the success of huDCIR-KI mouse generation.

Based on our previous findings that huDCIR expression in neutrophils and monocytes can be significantly induced during inflammatory perturbations, we evaluated huDCIR expression in a murine ZymD-induced peritonitis model and a dextran sodium sulfate–induced (DSS-induced) colitis model, where neutrophil and monocyte activation and infiltration are evident during the disease phase (28, 29). Consistent with our previous findings, we observed a robust induction of huDCIR^+^ cells in the peritoneal fluid during peritonitis (Figure 4, F and G) and in the colon crypts during DSS-induced colitis of huDCIR-KI mice (Figure 4, H and I, and Supplemental Videos 1 and 2). In agreement with our H&E staining results on human tissues, few DCIR^+^ cells were detected in the disease-free state. These results demonstrate the translational potential of our huDCIR-KI mouse for developing huDCIR-based therapies.

### Agonistic anti-DCIR mAb ameliorates experimental acute peritonitis.

**Figure 5:**
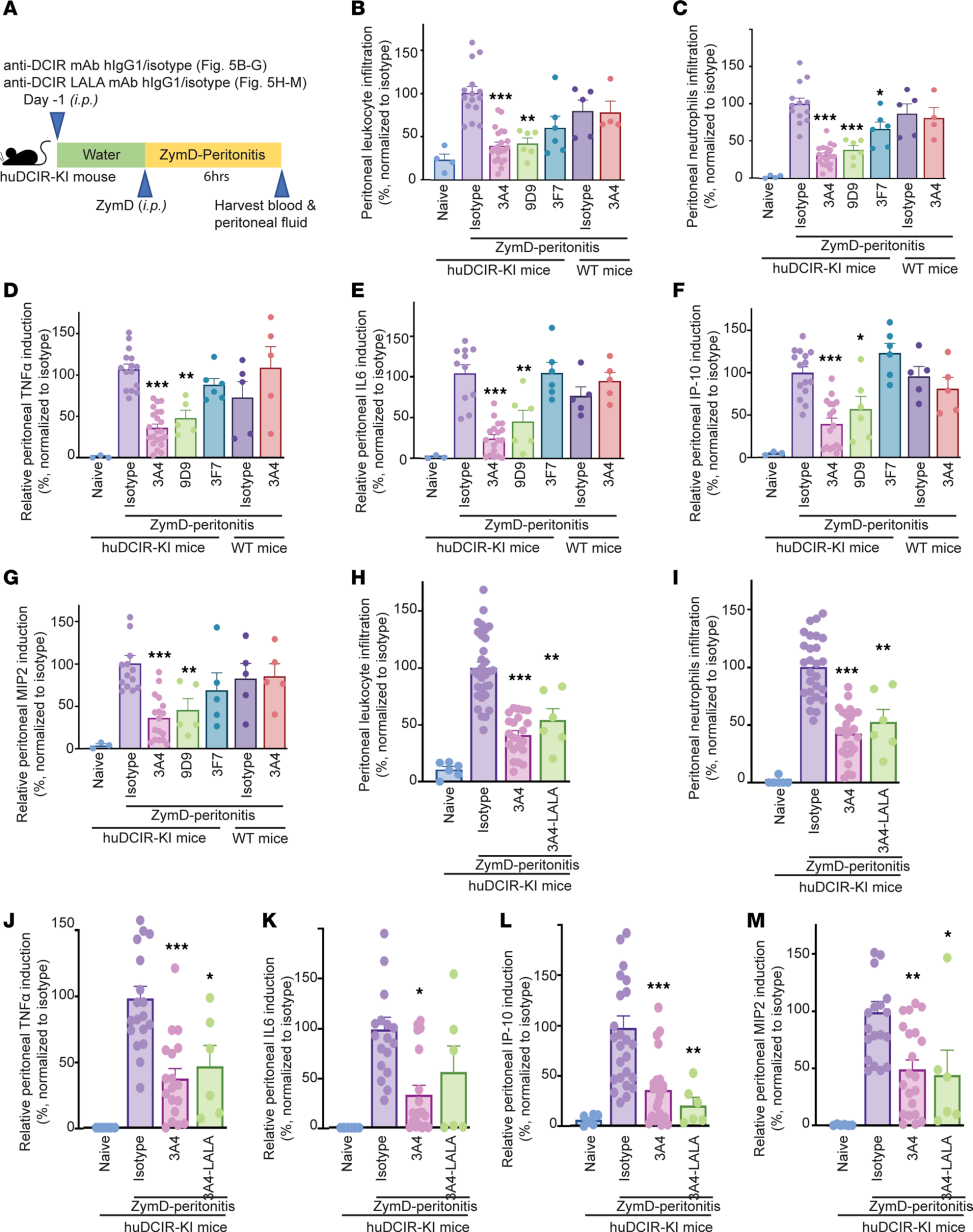
Agonistic anti-DCIR mAb ameliorates experimental acute peritonitis. (**A**) Study design of the prophylactic anti-DCIR mAbs treatment in the ZymD-induced peritonitis model. Anti-DCIR mAbs and Fc-matched isotype control were i.p. injected into the mice 1 day before the i.p. administration of 0.5 mL 2 mg/mL ZymD to induce peritonitis. (**B**–**G**) Relative leukocyte and neutrophil infiltration (**B** and **C**) and cytokine production in the peritoneal lavage (**D**–**G**) collected from the mice received i.p. administration of 10 mpk anti-DCIR mAbs clone 9D9 (*n* = 6 huDCIR-KI mice), 3A4 (*n* = 4 WT or *n* = 22 huDCIR-KI mice), 3F7 (*n* = 6 huDCIR-KI mice), or isotype (*n* = 5 WT or 14 huDCIR-KI mice) in the peritonitis model as described in **A**. (**H**–**M**) Relative leukocytes and neutrophils infiltration (**H** and **I**) and cytokine production in the peritoneal lavage (**J**–**M**) isolated from the mice received 10 mpk i.p. administration of agonistic anti-DCIR mAb clone 3A4 with WT (*n* = 22) or LALA (L234A and L235A) mutant huIgG1 Fc (*n* = 6) or isotype (*n* = 31) in the peritonitis model as described in **A**. Data are normalized to the isotype group. Each datum represents an individual mouse treated with indicated condition. Means ± SEM are shown, and statistical significance is determined by 1-way ANOVA test with Dunnett’s correction for multiple comparison to the isotype treated condition. *P < 0.05, **P < 0.01, ***P < 0.001.

To investigate the antiinflammatory function of DCIR, we assessed the efficacy of agonistic and nonagonistic mAbs in a ZymD-induced peritonitis model (Figure 5A). Prophylactic treatment with the agonistic clones 3A4 and 9D9 significantly reduced the severity of peritonitis in huDCIR-KI mice, as indicated by the reduced accumulation of leukocytes and neutrophils in the peritoneal lavage fluid, compared with the Fc-matched isotype control treatment group. We did not observe significant effects for the agonistic clone 3A4 treatment in WT mice (Figure 5, B and C). Although the nonagonistic clone 3F7 showed some inhibitory effect in reducing peritoneal leukocyte and neutrophil accumulation, only the agonistic clones significantly suppressed cytokine production in the peritoneal fluid (Figure 5, D–G) and serum (Supplemental Figure 5, A–C). Since human IgG1 binds to mouse Fc receptors with lower affinity, our anti-DCIR mAbs might still modulate cell activation through binding to the ITIM- and ITAM-containing Fc receptors. To eliminate the influence of the antibody’s Fc portion in evaluating the agonistic effects, we compared WT 3A4 with a LALA (L234A and L235A) mutated 3A4 variant with compromised FcR-binding ability (30). The results indicate that the protective effect from strong agonism on DCIR is more dominant compared with the FcR binding, as there were no significant differences in peritoneal cell accumulation (Figure 5, H and I) or cytokine secretion in the peritoneal fluid and serum (Figure 5, J–M, and Supplemental Figure 5, D–G) between the WT and LALA-mutated 3A4 treatments. However, we cannot completely rule out the importance of the Fc portion without thoroughly evaluating the antibody-mediated cell clearance effect.

### Anti-DCIR antibody promotes neutrophil clearance via antibody-dependent cellular phagocytosis (ADCP) and cytotoxicity (ADCC).

The engagement of agonistic anti-DCIR mAb initiates immune inhibitory signaling that effectively suppresses the inflammatory response. However, it remains unclear whether the Fc-mediated ADCP and ADCC also contribute to resolving inflammation. Given that DCIR is highly expressed on activated neutrophils and other myeloid cells, ADCP and ADCC could facilitate the clearance of inflammatory cells and prevent chronic inflammation. Therefore, we sought to investigate whether the Fc fragment of anti-DCIR mAb promotes the resolution of inflammation.

**Figure 6:**
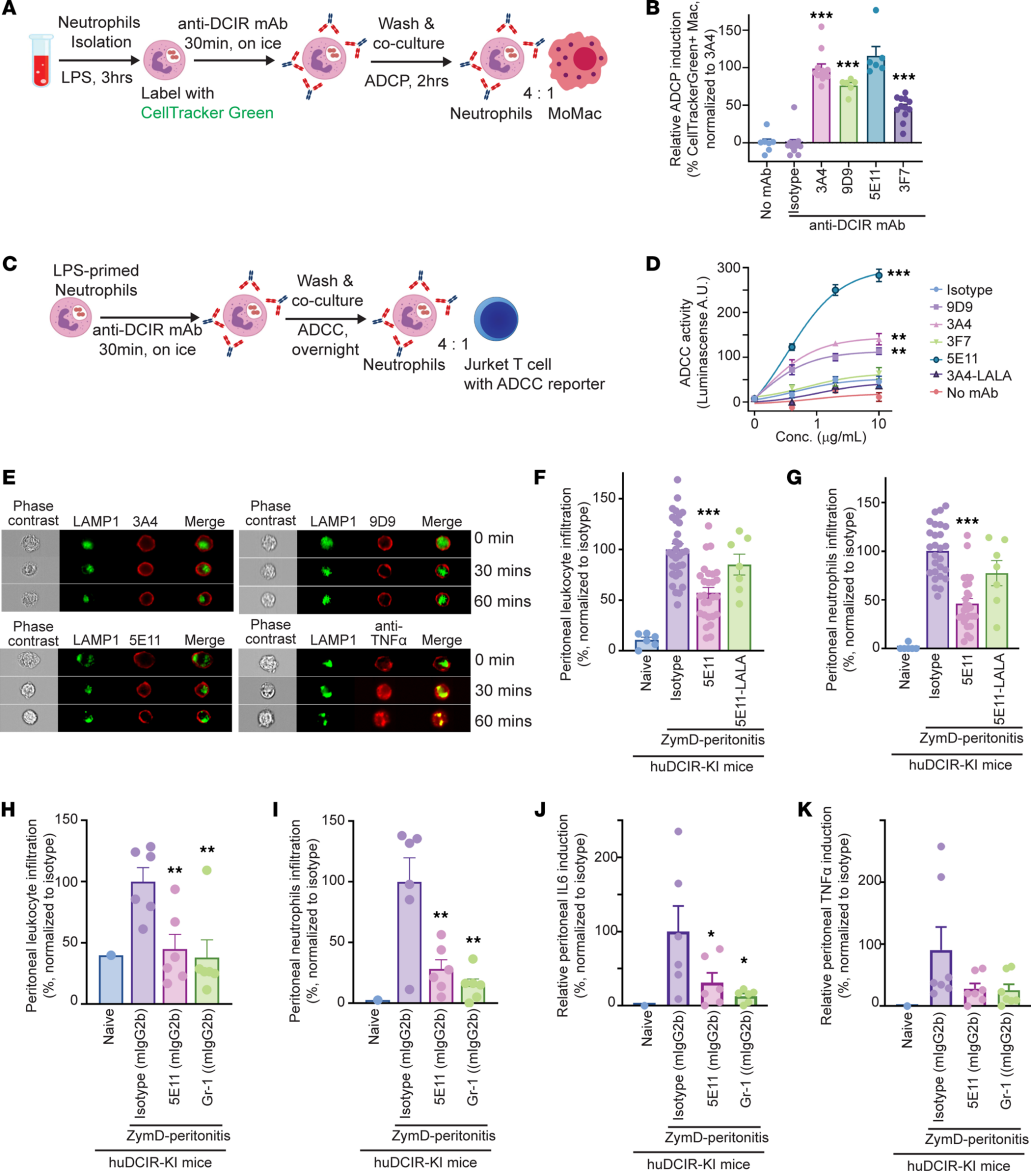
Anti-DCIR mAb promotes neutrophil clearance through ADCP and ADCC. (**A**) LPS primed human neutrophils were labeled CellTracker Green. Neutrophils were incubated with 5 μg/mL anti-DCIR mAbs (3A4, 9D9, 5E11, 3F7) or isotype for 30 minutes on ice. Neutrophils and monocyte-derived macrophages were cocultured (ratio 4:1). (**B**) ADCP were quantitated by flow cytometry detecting CellTracker Green^+^CD11b^+^CD66b^–^HLA-DR^+^macrophage after 2 hours coculture with 3A4 (*n* = 12), 9D9 (*n* = 6), 5E11 (*n* = 6), 3F7(*n* = 12), or isotype (*n* = 11). Data are normalized to 3A4 group. (**C**) LPS primed neutrophils were incubated with 3A4, 9D9, 5E11, 3F7, or isotype for 30 minutes on ice. Neutrophils and Jurkat cells containing an ADCC reporter were cocultured (ratio 4:1). (**D**) ADCC were quantitated by luminescence overnight. Representative data from 2 studies are shown. (**E**) Confocal flow cytometry of human monocytes treated with 10 μg/mL A647-labeled antibodies (red) for 0, 15, and 30 minutes at 37°C. Antibody internalization was determined by colocalization (yellow) of A647-labeled antibodies in lysosome, marked by A488 anti-LAMP1 antibody (green). (**F** and **G**) HuDCIR-KI mice received i.p. administration of 10 mpk 5E11 with WT (*n* = 25) or LALA mutant (*n* = 7) huIgG1 Fc or isotype (*n* = 25) 1 day before the i.p. injection of 0.5 mL 2 mg/mL ZymD. Relative leukocytes and neutrophils induction in peritoneal lavage were quantitated 6 hours after the ZymD injection. Data are normalized to isotype group. (**H**–**K**) Relative leukocytes and neutrophils infiltration (**H** and **I**) and cytokine in peritoneal fluid (**J** and **K**) collected 6 hours after ZymD injection, from mice pretreated with 5E11 with mouse IgG2b (5E11-mIgG2b) (*n* = 5), Fc-matched isotype (iso-mIgG2b) (*n* = 5), or anti–Gr-1 mAb (RB6-8C5) (*n* = 5) 1 day before the peritonitis. Data are normalized to isotype group. Each dot represents 1 biological replicate. Means ± SEM are shown, and statistical significance is determined by 1-way ANOVA with Dunnett’s correction compared with the isotype condition. *P < 0.05, **P < 0.01, ***P < 0.001.

To investigate whether anti-DCIR mAbs induce ADCP, we labeled LPS-primed neutrophils with CellTracker Green dye and cocultured them with human monocyte–derived macrophages in the presence of a panel of anti-DCIR mAbs or Fc-matched isotype control (Figure 6A). We found that both the agonistic clones 3A4 and 9D9 — as well as the weak-agonistic clone 5E11 and, to a lesser extent, nonagonistic 3F7 — displayed an enhanced ADCP effect, as indicated by the increased proportion of CellTracker Green^+^ macrophages (Figure 6B and Supplemental Figure 6). Moreover, using a commercial ADCC reporter system (Figure 6C), we found that 3A4, 9D9, and 5E11 can promote neutrophil clearance via Fc-mediated ADCC as well. However, the 3A4-LALA mutant failed to trigger the ADCC-associated signaling (Figure 6D).

Internalization of the cell surface target can impair the clinical benefit of ADCC-dependent therapeutic antibodies (31). Lectin receptors, such as CD209 and CD206, have been reported to promote antigen uptake through endocytosis (32, 33). Similarly, DCIR, as a C-type lectin, can also be endocytosed, resulting in reduced ADCC-mediated cell clearance. To assess the potential internalization effects of our humanized anti-DCIR mAbs, we compared the ADCC-inducing clones (3A4, 9D9, and 5E11) with an anti–TNF-α mAb after their binding to the overnight LPS-primed human MoDCs for up to 1 hour. Consistent with our previous finding (34), anti–TNF-α mAb binding to membrane TNF-α can trigger rapid internalization, indicated by the increased overlap between the AF647-labeled anti–TNF-α mAb and AF488-stained LAMP1, an endosome marker. However, we did not observe obvious endocytosis of anti-DCIR mAb, as most AF647-labeled anti-DCIR mAbs still localized on the plasma membrane (Figure 6E). These data suggest that our anti-DCIR mAb can provide an approach to overcome the endocytosis of DCIR and promote the antibody-mediated clearance of inflammatory DCIR^+^ cells.

To assess the in vivo antibody-mediated clearance of neutrophils, we compared the effects of WT and LALA mutant anti-DCIR mAbs in a ZymD-induced peritonitis mouse model (Figure 5A). To diminish the confounding effects of inhibitory signaling via the DCIR receptor, we i.p. injected the mice with a weak-agonistic clone 5E11, which significantly promotes both ADCP and ADCC effects. We observed a significant reduction in leukocyte and neutrophil accumulation in the peritoneal fluid of mice treated with WT clone 5E11, compared with the Fc-matched isotype control group. However, the introduction of the LALA mutation into this clone (5E11-LALA) compromised its ability to suppress peritoneal cell accumulation (Figure 6, F and G).

To fully exploit the ADCP and ADCC effects mediated by mouse Fc receptors CD16 and CD32, which are only partially activated by the human IgG1 Fc portion of our anti-DCIR mAbs due to suboptimal binding affinity (35), we generated an anti-DCIR mAb, clone 5E11, with mouse IgG2b (5E11-mIgG2b) and evaluated its efficacy in the ZymD-induced peritonitis model. Compared with the Fc-matched isotype control, 5E11-mIgG2b significantly reduced peritoneal leukocyte and neutrophil accumulation to a level comparable with that achieved by anti–Gr-1 mAb treatment, a standard approach for depleting mouse neutrophils (Figure 6, H and I). Consistent with the reduction in neutrophils, 5E11-mIgG2b treatment also suppressed production of proinflammatory cytokines IL-6 and TNF-α in the peritoneal lavage (Figure 6, J and K). Unfortunately, nonagonistic mAbs like 1G3 and 3F7 fail to induce robust ADCC effects, hindering our ability to dissect the distinct contributions of the Fab versus Fc portion separately, and 5E11 still exhibits a weak-agonistic effect as depicted in Figure 2C. In summary, by employing a potent agonistic mAb with abrogated Fc (3A4-LALA) and a weaker agonistic mAb (5E11) with potent ADCP/ADCC effects, we deduce that both the Fab and Fc portion contribute to the protective effects in our peritonitis model.

### Agonistic anti-DCIR mAb attenuates the experimental colitis.

**Figure 7:**
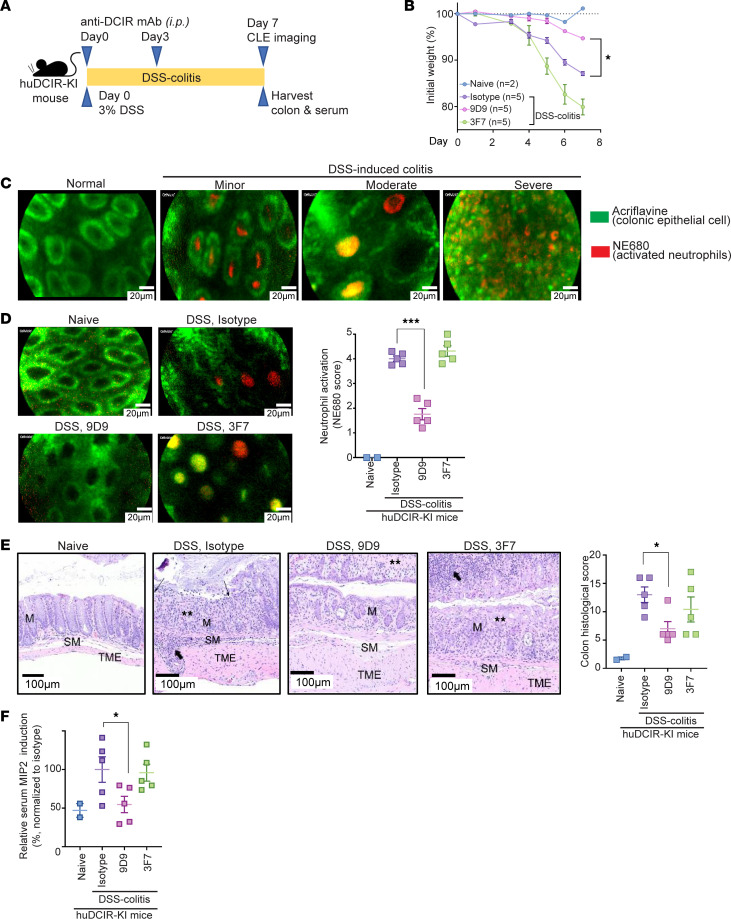
Administration of agonistic anti-DCIR mAb attenuates experimental colitis. (**A**) Study design of murine DSS-induced colitis model with prophylactic anti-DCIR mAbs treatment. huDCIR-KI mice were i.p. injected with 10 mpk anti-DCIR mAbs (9D9, 3F7) or isotype control (*n* = 5/antibody-treated group) on day 0 and 3 during the 7 days long 3%DSS water feeding period. (**B**) Body weight change of the mice treated with indicated antibodies during the DSS-colitis phase. Weight is presented relative to the initial body weight before DSS-water feeding. (**C**) Representative confocal laser endoscopy (CLE) image of the colonic crypts (green, labeled by Acriflavine) with infiltrated inflammatory neutrophils (red, labeled by NE680) from mice received 3%DSS for 7 days with different colitis severity. (**D** and **E**) Representative CLE image of colonic crypts with associated neutrophil activation score indicated by the NE680 staining (**D**), and representative H&E-stained colons (**E**) with associated histology score of the mice received anti-DCIR mAbs and isotype control treatment as illustrated in **A**. Scale bars: 20 μm for **D** and 100 μm for **E**. M, mucosal gland; SM, submucosa; TME, tunica muscularis externa; thin arrows, erosion; thick arrows, inflammatory cells; **, gland loss. (**F**) MIP-2 cytokine level in mice serum collected on day7 after the DSS-colitis. Data are normalized to isotype group. Each data point represents the individual mouse treated with indicated condition. Means ± SEM are shown, and statistical significance is determined by 1-way ANOVA test with Dunnett’s correction for multiple comparison to the isotype treated condition. *P < 0.05, ***P < 0.001.

Our results suggest that engagement of agonistic anti-DCIR mAb triggers immune inhibitory signaling, providing a suppressive effect; meanwhile, the Fc fragment of anti-DCIR mAb facilitates clearance of inflammatory cells, promoting resolution of inflammation. These findings support the potential use of anti-DCIR mAbs as therapeutic agents for chronic inflammatory diseases such as IBD. To evaluate this potential, we treated mice with agonistic anti-DCIR mAb (9D9), nonagonistic anti-DCIR mAb (3F7), or isotype control prior to (on day 0) and during (on day 3) DSS exposure (Figure 7A). Administration of agonistic anti-DCIR mAb significantly ameliorated DSS-induced colitis, indicated by less body weight loss (Figure 7B). We have found that the extent of neutrophil activation and crypt abscesses correlates with the disease severity in the DSS-induced colitis model by confocal laser endoscopy (CLE) (Figure 7C). Since huDCIR highly expresses on neutrophils accumulated in the colon crypts of huDCIR-KI mice during DSS-induced colitis (Figure 4I), we analyzed the infiltrated neutrophil activation and the integrity of crypts by CLE and found that administration of agonistic anti-DCIR mAb (9D9) decreased neutrophil elastase staining (NE680) and increased integrity of crypts (Figure 7D and Supplemental Videos 3–6). In the histology of colon, agonistic antibody (9D9) treatment reduced erosion and inflammatory cell infiltration with less hyperplastic and expanded mucosal glands and submucosa compared with nonagonistic anti-DCIR mAb (3F7) and isotype control treatments (Figure 7E). In addition, agonistic antibody (9D9) significantly reduced the MIP-2 secretion (Figure 7F) compared with nonagonistic anti-DCIR mAb (3F7) and isotype control treatments, indicating a reduced chemotaxis to the source of inflammation and activation of neutrophils. Overall, administration of agonistic anti-DCIR mAb significantly ameliorated DSS-induced colitis.

## Discussion

Immunoinhibitory receptors play a crucial role in maintaining immune homeostasis by regulating excessive immune responses and preventing the development of inflammatory diseases. As a result, targeting these receptors with antagonist antibodies has emerged as a widely adopted approach for cancer treatment. However, utilization of agonistic antibodies to restore immune tolerance in inflammatory diseases remains poorly understood. In this study, we investigated the effects of DCIR agonistic antibodies on immune signaling and discovered their ability to trigger immunosuppressive pathways. Specifically, we found that DCIR agonistic antibodies recruit SHP2 to the intracellular ITIM domain, thereby inhibiting the activation of the immune-stimulating SHP2-SYK pathway that is typically initiated by other proinflammatory pattern recognition receptors.

SHP2 can function as an immunosuppressor by dephosphorylating and inactivating downstream molecules involved in the proinflammatory signaling pathways. For instance, SHP2 can dephosphorylate TCRζ chains, ZAP70, and the costimulatory receptor CD28, thereby preventing T cell activation and reducing the production of proinflammatory cytokines (22–24). Interestingly, other studies suggest that the phosphatase activity of SHP2 is not essential for immune checkpoint receptor function (16), indicating the involvement of additional regulatory mechanisms beyond its enzymatic activity. Additionally, SHP2 has been reported to act as an adaptor protein that mediates protein-protein interactions, such as ITAM-SHP2-SYK interaction, thereby promoting the activation of immune pathways (25). Hence, even elevated SHP2 expression has been observed in various autoimmune diseases (36–38). SHP2 can be hijacked to facilitate proinflammatory signaling when strong agonists of inhibitory receptors are absent. Our study provides proof of concept that agonistic signaling through ITIM-bearing DCIR can compete with ITAM-containing pattern recognition receptors for SHP2 binding and restrain proinflammatory signaling.

Given its lectin-like properties, DCIR can recognize various glycoproteins, suggesting a potential overlap in antigens recognized by other lectin receptors (21). It is plausible that DCIR might form coinhibitory complexes through cross-linking with these shared antigens. Notably, previous studies have shown that cross-linking multiple immune inhibitory receptors using multivalent antibodies can lead to a more robust suppressive effect compared with targeting individual receptors (39–41). Although we confirmed that our agonistic anti-DCIR antibody can further amplify DCIR signaling even when it is occupied by its natural ligands, such as mannose and NA2-glycan, our study did not directly investigate the effect of our agonistic anti-DCIR antibody on the binding of DCIR to other unknown ligands or DCIR’s potential cross-linking with other immune receptors. We acknowledge the possibility that our agonistic antibody may disrupt the formation of coinhibitory complexes in the presence of DCIR’s natural ligands. To address this limitation, future studies can explore the application of a bispecific agonistic antibody design to cluster DCIR with another immune inhibitory receptor, such as PD-1, or utilize engineered Fc regions to enhance cross-linking with FcγIIB. These strategies have potential to significantly improve the immune regulatory function by strengthening the coinhibitory complex.

In addition to its agonistic effect, our anti-DCIR antibody may promote inflammation resolution through Fc-mediated neutrophil clearance. While neutrophils play a critical role in antimicrobial defense, they can also contribute to tissue damage by releasing proinflammatory factors such as ROS, granule proteases, and cytokines. Efficient clearance of tissue-infiltrated neutrophils by phagocytes is essential for the prevention of chronic inflammation. Multiple mechanisms have been reported for inducing immunotolerant phagocytes. One such mechanism involves lipid metabolism initiated by engulfed apoptotic cells, which activates β-oxidation and the translocation of PPARs and LXR/RXR. Activation of these pathways can upregulate proresolving factors like IL-10 and TGF-β (42, 43). Accelerating neutrophil clearance not only prevents the systemic release of proinflammatory ligands and the formation of neutrophil extracellular traps (NETs), but it also supports the development of tolerogenic macrophages. Therefore, agonistic anti-DCIR antibodies could provide therapeutic benefits for chronic diseases driven by neutrophil-mediated pathology, including chronic obstructive pulmonary disease (COPD), neutrophilic asthma, RA, and gouty arthritis.

Inconsistencies in *DCIR^–/–^* mouse phenotypes during DSS-induced colitis have been noted, with Sun et al. (44) reporting protection and Hütter et al. (45) indicating increased susceptibility. This variability suggests potential influences of environmental factors, such as commensal microbes and diet, on muDCIR’s natural ligands, altering gut glycan structure and affecting native agonistic effects (46–48). Sun’s study attributes reduced MIP-2 to increased GM-CSF from ILC3 in response to excessive IL-1b secreted by hyperactivated *DCIR^–/–^* macrophages, suggesting an indirect effect (44). Our investigation, using huDCIR-KI mice and potent agonistic mAbs, consistently reveals reduced MIP-2 and neutrophil accumulation in peritonitis and DSS-colitis models. This supports our hypothesis and is in agreement with our findings that potent antibody agonism may overcome the weak effects seen with natural ligands. Notably, differences between mouse and human DCIR could contribute to this variation, underscoring the need to explore huDCIR biology in humanized models. Our huDCIR-KI mice and anti-huDCIR mAbs offer valuable tools for translational animal studies supporting drug development. Nevertheless, we acknowledge that our prophylactic drug dosing strategy may be less applicable to clinical practices. The acute nature of the peritonitis and colitis models we utilized, with rapid kinetics for neutrophil and monocyte/macrophage activation, limits the testing window for our anti-DCIR mAbs once the disease has developed. Employing appropriate animal models that represent chronic diseases driven by neutrophils and monocytes/macrophages-mediated pathology, such as COPD and neutrophilic asthma, would yield more informative results for examining the therapeutic effects of targeting DCIR.

## Methods

### Sex as a biological variable.

For studies involving mice models, male and female mice were studied with no clear differences discerned by sex. Results from the male and female mice were pooled.

For studies involving human tissues and PBMC, male and female samples were pooled for analysis, and sex was not considered as a biological variable.

### Bioinformatic analysis of DCIR expression.

For the scRNA-Seq study, scRNA-Seq data of patients with HS and CD were extracted from GSE155850 (49) and GSE134809 (50), respectively. BioTuring was used to analyze DCIR expression from HS lesional skin samples and CD ileum tissue. Differential expression analysis (DEA) was performed using BioTuring software (51) with Venice method (52) comparing lesion and normal tissue.

For bulk RNA-Seq study in human PBMC, DCIR mRNA fragments per kilobase of exon per million mapped reads (FPKM) were extracted from the BLUEPRINT data set (www.blueprint-epigenome.eu). For the IBD bulk RNA-Seq data set, DCIR comparative expression was analyzed based on the raw data extracted from GSE16879 (53). Log_2_ transformed fold change of DCIR expression in each condition was shown compared with the normal control. For the other bulk RNA-Seq data set, data were analyzed using Omicsoft Array Studio v12.0 (Qiagen).

### IHC staining.

Disease tissues from the skin of HS and SLE and mucosal biopsy of CD were collected in 10% formalin. Tissue sections were prepared from paraffin blocks and stained with H&E, anti-DCIR antibody (MilliporeSigma, HPA007842), and rabbit IgG isotype control (MilliporeSigma, SAB5500149).

### Generation and characterization of anti-huDCIR mAbs.

Sprague Dawley JR14 rats were immunized with a vector expressing full-length huDCIR cDNA using a gene gun. Splenic B cells from immunized rats were used for hybridoma preparations. The supernatants from hybridomas were screened based on their ability to bind to huDCIR-transfected HEK293 cell and human MoDCs by FACS. Hybridomas producing strong huDCIR binder were selected for the generation of anti-DCIR mAb. The heavy and light chain variable regions (VH and VL) of the hybridomas were cloned into a human IgG1 backbone and expressed as chimeric mAbs, and their cross-reactivity to cyno DCIR and huDCIR — but not muDCIR1 — was confirmed. Fully humanized antibodies were produced after removal of rat-derived sequences and codon optimization. The resultant humanized anti-DCIR mAbs produced from the Expi293 cells were purified using the IgG affinity columns with protein A resin. Purified antibodies were evaluated for thermal stability and monomer content (>96% monomer). Subsequently, the humanized antibodies were evaluated for the species’ cross-reactivity to huDCIR, cyno DCIR, or muDCIR1 expressing HEK293 cells (Supplemental Table 1). Selected antibodies were also evaluated for cross-reactivity to other CLRs and pattern recognition receptors, including L-SIGN, DC-SIGN, DNGR-1, Dectin-1, CD123, CD205, CD206, CD207, and CD301. The LALA antibody mutants were generated as described by the Winter group (30), involving replacement of leucine (L) with alanine (A) at positions 234 and 235 of humanized antibodies.

### Generation of huDCIR agonistic signaling reporter cell line.

HuDCIR agonistic signaling reporter cell line was generated using the HEK293 cell carrying a huDCIR cDNA–expressing vector with an ITAM domain cloned from human DECTIN1 cDNA to replace the original ITIM domain (Supplemental Table 1). The resultant HEK293 cell was transfected with a second NF-κB luciferase reporter vector (*pGL4.32-NF-*κ*B Luc2P, Promega* E8491). The agonistic effect induced by the antibody was detected by luciferase activity employing a luminescence detection assay (Promega, E967-A/B). For testing DCIR’s ligand binding, 100 μL of mannose-BSA, NA2-BSA (synthesized by Biosynth) were coated to the high-binding tissue culture plate (Corning, 3361) overnight at 4°C. After descanting the unbound glycans, HEK293 cells with huDCIR agonistic signaling reporter were seeded in the plate. The agonistic effects induced by the ligands were detected by a luminescence detection assay. For evaluating the effect of glycan binding to the agonist effect of anti-DCIR antibody, mannose-BSA or NA2-BSA (50 μg/mL) were coated on the plate and cultured with the HEK293 cells containing a huDCIR agonistic signaling reporter for 6 hours. Agonistic anti-DCIR mAbs (9D9) were added for overnight culture. Natural glycan’s effects on the antibody’s agonistic effect were determined by the luciferase assay.

### Cell culture and stimulation.

Leukopaks and whole blood were obtained from Sanguine Biosciences. Untouched neutrophils were isolated from blood by negative selection (Miltenyi Biotec, 130-104-434). PBMCs were isolated from Leukopaks using Ficoll density gradient centrifugation (GE Healthcare). Blood cells were overlaid on Ficoll and centrifuged for 20 minutes at 600*g* with no brake. PBMCs were collected from the interphase layer. CD14^+^ monocytes were purified from PBMCs using microbeads (Miltenyi Biotec, 130-090-879). Human MoDCs were differentiated from monocytes supplemented with 20 ng/mL human IL-4 (Peprotech, 200-04) and 200 ng/mL human GM-CSF (Peprotech, 300-03) for 6 days. Human neutrophils, monocytes, and MoDCs were cultured in complete RPMI 1640 medium (Thermo Fisher Scientific, 11875093) containing 10% FBS (Cytiva, SH30071.03), GlutaMAX (Thermo Fisher Scientific, 35050079), NEAA (Thermo Fisher Scientific, 11140050), pyruvate (Thermo Fisher Scientific, 11360070), penicillin + streptomycin (Thermo Fisher Scientific, 15140122), 50 μM 2-mercaptoethanol (Thermo Fisher Scientific, 21985023), and 25 mM HEPES (Thermo Fisher Scientific, 15630080). For monocyte-derived macrophage differentiation, the monocytes were cultured in the complete DMEM (Thermo Fisher Scientific) supplemented with 50 ng/mL human M-CSF (Peprotech, 300-25) and 10% FBS (Thermo Fisher Scientific) for 7 days.

### Immune complex and zymosan stimulation.

To make anti–human IC: HSA, 2 mL of 5 mg/mL anti–human serum albumin IgG fraction (MPBio, 855029) and 1 mg of human albumin (MPBio, 191349) were mixed at 37°C for 30 minutes, centrifuged at 200*g* for 5 minutes to remove the unbound IgG in the supernatant, and resuspend in sterile 1 mL PBS. To induce the activation of FcRγ ITAM motif, cells were treated with 50 μg/mL immune complex. To evaluate the cytokines production from the activation of the ITAM containing Dectin-1 receptor, cells were stimulated with 25 μg/mL ZymD (InvivoGen, tlrl-zyd) overnight. Supernatant was collected for MSD assay measuring secreted human TNF-α, IL-6, IL-1β and IFN-γ.

### Neutrophile stimulation and oxidative burst analysis.

Neutrophils were incubated in 10 μg/mL anti-huDCIR mAbs or isotype-coated nontreated plates for 2 hours 37°C. Then, 1 × 10^5^ neutrophils were seeded on XFe96 Cell Culture Microplates (Agilent Technologies, 103794-100) coated with 22.4 μg/mL Cell-Tak (Corning Inc., 354240) in XF medium and incubated in a non-CO_2_, 37°C incubator for 45 minutes. GM-CSF (100 ng/mL) and PMA (100 ng/mL) were added to injection ports A and B of the sensor cartridge (Agilent Technologies, 103792-100). Neutrophil oxidative burst was analyzed by the Seahorse XFe96 Analyzer through the measurement of OCR. The AUC of the OCR was calculated by GraphPad Prism software.

### Flow cytometry.

Cells were blocked with 5% human AB serum (MilliporeSigma, 4522) or rat anti-mouse CD16/CD32 antibody (BD Biosciences, 553142) for 30 minutes before mixed with antibodies and incubated on ice in the dark for 30 minutes. Blood or homogenized tissue cells were lysed and fixed before FACS analysis using Fix/Lyse Solution (Invitrogen, 00-5333-54). Samples were acquired on a FACS Canto II cytometer (BD Biosciences), and analysis was performed using Flowjo software. Antibodies used for FACS analysis include PE-Cy7 anti–human CD11b (101216, BioLegend), APC anti–human CD66b (392912, BioLegend), APC-Cy7 anti–human HLA-DR (307618, BioLegend), APC anti–human CD16 (302012, BioLegend), FITC anti–human CD14 (325603, BioLegend), APC-Cy7 anti–human CD3 (300318, BioLegend), BV510 anti–human CD19 (302242, BioLegend), AF488 anti–mouse Ly-6G (127626, BioLegend), PE anti–human DCIR (FAB1748P, R&D system), PE rat anti–mouse DCIR1 (Clec4a2) (566810, BD Bioscience), APC anti–human IgG (H+L) (109-136-088, Jackson ImmunoResearch), and PE anti–human IgG Fcγ fragment specific ((09-115-098, Jackson ImmunoResearch).

### Immunoprecipitation and Western blot (WB).

Cells were lysed in cold IP lysis buffer (Thermo Fisher Scientific, 87788) supplemented with protease and phosphatase inhibitors (Thermo Fisher Scientific, 78444). Cell debris was cleaned by centrifuging at 21,000*g* for 15 minutes, and the supernatant was used for WB or immunoprecipitation. Target protein was pulled down by incubating 100 μL cell lysate with 1 μg antibody at 4°C overnight, followed by adding 25 μL Dynabeads M-280 (Thermo Fisher Scientific, 11204D) at room temperature and rotating for 30 minutes. After 4 washes with cold TBST buffer (1× TBS, 0.1% Tween-20), proteins were eluted with 2× SDS loading buffer and boiled for 10 minutes. The resultant samples were separated by SDS-PAGE and probed with indicated antibodies. Antibodies used for immunoprecipitation and WB includes anti–p-Tyr (Cell Signaling Technology, 8954S), anti–β-actin (Cell Signaling Technology, 12620), anti-DCIR (clone 1G3), anti-SHP2 (Cell Signaling Technology, 3397S), anti-Syk (BioLegend, 644302), and anti-FcRγ chain (MilliporeSigma, 06-727; Cell Signaling Technology, 20379S). HRP-conjugated goat anti–rabbit IgG (Novus, NB7187) and goat anti–mouse IgG (Novus, NB7574) were used as secondary antibodies.

### ADCP and ADCC assay.

For measuring ADCP, human neutrophils were pretreated with 100 ng/mL of LPS (tlrl-peklps, InvivoGen) for 3 hours and labeled with CellTracker Green (C2925, Thermo Fisher Scientific). The dyed cells were treated with indicated concentrations of antibodies on ice for 30 minutes and washed with cold PBS to remove unbound antibodies; they were then cocultured with monocyte-derived macrophages at 37°C for 2 hours. The ratio between neutrophils and macrophages was 4:1. The phagocytosis score was evaluated based on the FACS analysis for the percentage of CellTracker Green^+^ macrophages (CD66b^–^CD11b^+^HLA-DR^+^). For measuring ADCC, antibodies bound and LPS primed neutrophils were cocultured with Jurkat T cells containing an ADCC reporter (jktl-nfat-cd16, Invivogen) at 37°C overnight. ADCC effect was analyzed per vendor’s protocol. The ratio between neutrophils and Jurkat T cells was 4:1.

### Antibody internalization assay.

Antibodies were labeled with AF647 using Labeling Kit (Invitrogen, A20186) per manufacturer’s protocol. Human MoDCs were blocked with 5% human AB serum (MilliporeSigma, H4522) for 30 minutes and incubated with 1 μg/mL A647 antibodies for 15 minutes on ice. After washing with cold PBS, cells were cultured at 37°C for 0, 30, 60 and 120 minutes. The cells were fixated and permeabilized using ice cold Fix/Perm buffer (BioLegend, 426803). The internalized mAbs were identified by an Amnis ImageStream X Mk II confocal flow cytometry imaging system (Luminex). Traffic of internalized mAbs to lysosome were determined by colocalization with LAMP1 detected by the A488 anti–human LAMP1 antibody (BioLegend, 328610).

### huDCIR-KI mice generation and genotyping.

huDCIR KI mice were generated at GenOway Inc. using a target construct, containing huDCIR cDNA adjacent to an IRES-tdTomato cassette, next to a *loxP*-flanked neomycin resistance cassette, inserted in the *muDCIR1* (*Clec4a2*) exon 4. Targeted ES cells with recombinant DCIR-tdTomato-NeoR locus were injected into blastocysts, followed by embryo transfer to the pseudo pregnant female C57BL/6 albino foster mice. The resulting male chimeras were mated with female Cre-deleted mice on a C57BL/6 background to create HET huDCIR-KI mice. The HET huDCIR-KI mice were then bred with WT C57BL/6 mice for at least 7 generations before being inbred with the HET to get HO huDCIR-KI mouse. Genomic DNA from tail snipping was genotyped by Southern blot analysis. EcoRV-digested genomic DNA were separated by 1% agarose gel electrophoresis and transferred to the nitrocellulose membranes. A radioactive labeled probe with complementary sequence to the *muDCIR1* was incubated with the membrane for hybridization. After hybridization, the unhybridized probe was removed by washing in SSPE buffer. DNA fragments bound with radiolabeled probes were detected using x-ray film, resulting in a 6.1 kb band for the WT or a 9.3 kb band for the huDCIR-KI within the target locus.

### Zymosan-induced peritonitis model.

Mice were i.p. injected with 0.5 mL of 2 mg/mL ZymD (InvivoGen, tlrl-zyd) solubilized in low-endotoxin sterile PBS. Mice were anesthetized with isoflurane and the peritoneal cavity lavage fluid was collected at 6 hours after injection. Peritoneal lavage cells were characterized and quantitated using a hematology analyzer (Sysmex Inc.). The peritoneal lavage was spun at 500*g* for 10 minutes to collect the supernatant for cytokine measurement. Terminal blood was collected into EDTA tubes and spun at 2,000*g* for 10 minutes to acquire the serum for cytokine measurement. Mean of leukocytes or neutrophils absolute numbers identified from the mice with peritonitis and isotype control treatment was set as benchmark (100%). Leukocyte or neutrophil numbers from anti-DCIR mAb–treated mice were divided by the mean of leukocyte or neutrophils from mice treated with isotype control to reflect the percent reduction of infiltrated cell due to the drug treatment.

### DSS-induced colitis model.

Colitis was initiated by feeding mice with 3% DSS (MP Biomedicals, 02160110-CF) in autoclaved drinking water ad libitum for 7 days.

Infiltrating DCIR^+^ cells and vascular permeability in the distal colon were evaluated using CLE (CellVizio Dual Band, Mauna Kea Technologies) imaging. Mice were i.v. administrated with 100 μL of 1 mg/mL 10 kDa AF680 dextran (Thermo Fisher Scientific, D34680) 2 days before feeding DSS water. Antibodies were labeled with AF488 using labeling kit (Invitrogen, A10235) per manufacturer’s protocol and i.v. injected into mice on day 6 after the DSS treatment. CLE was performed on day 0 for naive and day 7 for colitis mice.

For DCIR Ab administration, mice were weighed and randomized into groups. The treatment groups received 10 mpk of antibodies through i.p. on day 0 and day 3. Mice were weighted daily until the end of the experiment. The CLE analysis was done according to our previous publication (54). Prior to CLE examination on day 7, mice were i.v. injected 4 nM of NE680 (PerkinElmer, NEV11169). Four hours later, mice were anesthetized under continuous oxygen/isoflurane. Colon was washed with saline, followed by intrarectally stained with 200 μL of 0.1 % acriflavine (Sigma-Aldrich, A8126). The 2 cm distal colon was captured by CLE and analyzed. Each video was assessed and blindly scored based on NE680 and acriflavine staining (Supplemental Table 2). Mice colons were further isolated and fixed in 10% formalin for paraffin embedding. Samples were sectioned at 5 μm and stained with H&E. The distal 4 cm of colon was microscopically assessed for histological scoring (55).

### Statistics.

Statistical analysis was performed using GraphPad Prism software. Significance between 2 groups was determined by unpaired, 2-tailed Student’s *t* test, and significance between multiple groups was determined using 1-way ANOVA with Dunnett’s post hoc test. All composited and representative data are generated from at least 2 independent experiments.

### Study approval.

All animal procedures were approved by the IACUC of AbbVie Inc. under Protocol ID 36-II. Animal numbers were empirically determined to optimize numbers necessary for statistical significance based on previous reports utilizing same disease models. All human experiments were performed and samples were collected are under written informed consent and approval by the ethics committee of the corresponding hospital and AbbVie Inc.

### Data availability.

Single-cell RNA-Seq data of patients with HS and CD were downloaded from GSE155850 (49) and GSE134809 (50). Bulk RNA-Seq results were extracted from the BLUEPRINT data set (https://blueprint-epigenome.eu) and GSE16879 (53) for DCIR mRNA level in human PBMC and IBD tissues, respectively. Results for the metaanalysis of DCIR expression in patients with IBD, RA, or HS were generated from the nonpublic Qiagen Omicsoft Suite HumanDisease_B37 database, and raw data can be made available from the corresponding author upon request. Values for all data points in graphs are reported in the Supporting Data Values file.

## Author contributions

LC, SP, and HJW designed the experiments and wrote the manuscript with input from JB, FD, AD, TR, KW, SW, and GV. Most of the in vitro experiments and analyses were performed by LC, SP, JB, JW, NC, ED, LJ, and HJW. MP, SZ, SP, and FD led the antibody generation and characterization. DM and SL assisted LC, SP, and HJW with the animal studies. KO, GN, YY, and SW performed the histopathological analyses. All contributing authors discussed the results in the final manuscript and agreed to submit this manuscript for publication.

## Supplementary Material

Supplemental data

Unedited blot and gel images

Supplemental video 1

Supplemental video 2

Supplemental video 3

Supplemental video 4

Supplemental video 5

Supplemental video 6

Supporting data values

## Version 1 05/23/2024

In-Press Preview

## Version 2 06/24/2024

Electronic publication
