# Supplementary material for: Agonistic anti-DCIR antibody inhibits ITAM-mediated inflammatory signaling and promotes immune resolution
Source: JCI Insight. 2024 May 23;9(12):e176064. doi: 10.1172/jci.insight.176064 (PMC11383175; doi:10.1172/jci.insight.176064)
Supplement: Supplemental data [file jciinsight-9-176064-s071.pdf]

Supplementary Materials for

Agonistic anti-DCIR antibody inhibits ITAM-mediated inflammatory signaling and promotes immune resolution

Liang Chen *et al.*

Corresponding author: Hsi-Ju Wei, hsijunico.wei@AbbVie.com

The PDF file includes:

Figures: S1 to S6

Tables: S1 and S2

Movie legends: S1 to S6

Other Supplementary Material for this manuscript includes the following:

Movies: Video S1 to S6

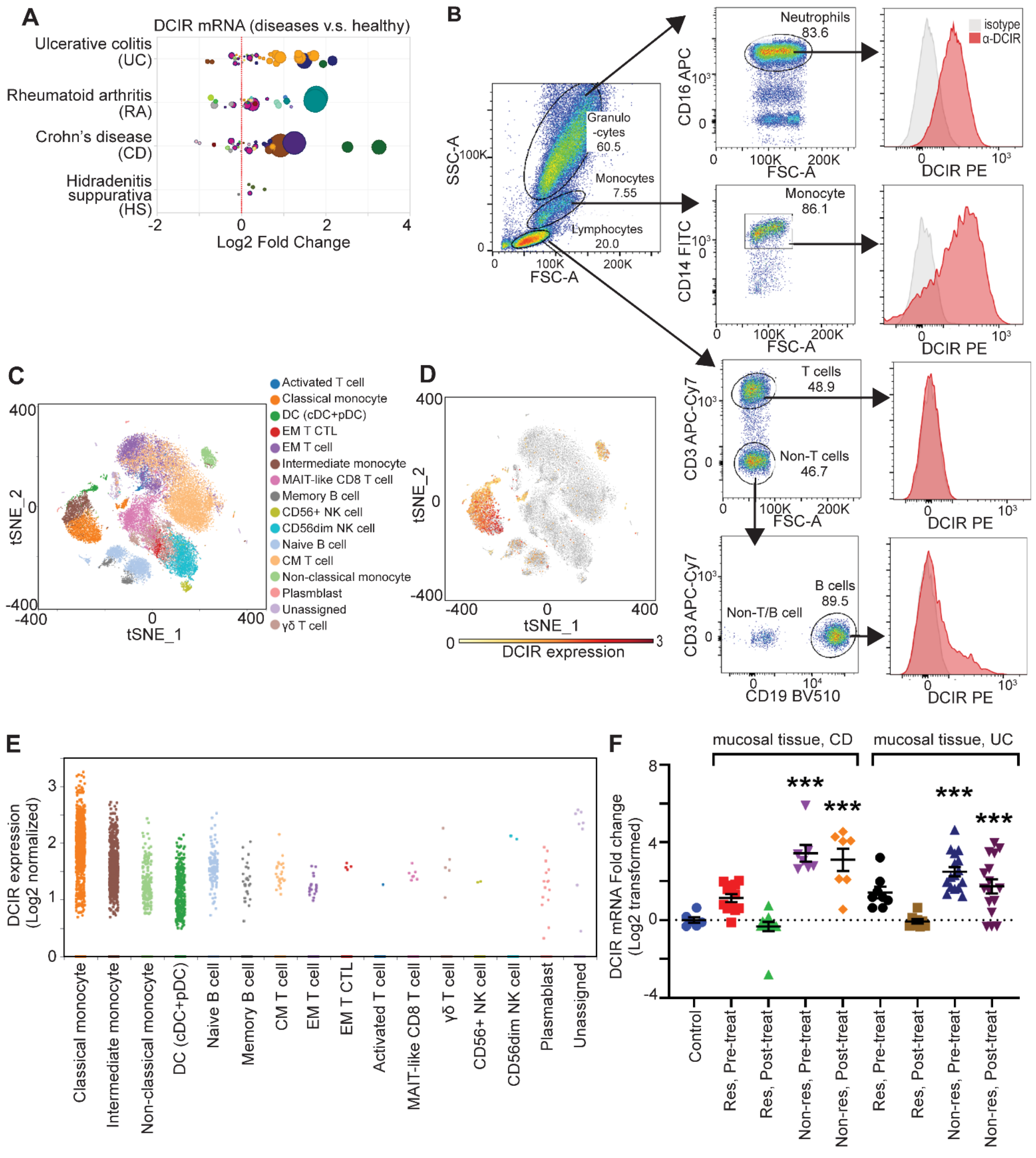

Supplementary Figure 1: Increased DCIR expression hallmarks the inflammatory stage of multiple autoimmune diseases.

(**A**) Comparative analysis of DCIR expression in disease v.s. normal tissues from UC, RA, CD, or HS patients (data source: Qiagen Omicsoft Suite HumanDisease_B37 database). The bubble plot shows DCIR mRNA fold change from comparing the indicated disease conditions to the healthy normal control. Each bubble's color and size indicate the different sampling tissue and p value, respectively. **(B)** Flow cytometry of DCIR expressed in the neutrophils, monocytes, T and B cells isolated from unperturbed human PBMCs. Representative data from 2 studies is shown. **(C)** t-SNE plot of single-cell RNA sequencing analysis for mucosal tissue collected from CD

patients (data source: GSE134809). **(D)** DCIR-expressing cells overlaying on identified cell clusters in the t-SNE plot from scRNA-sequencing analysis for CD patients. **(E)** DCIR mRNA level in different cell clusters were quantitated by pseudo-bulk differential expression analysis. **(F)** DCIR mRNA level in normal and disease mucosal tissues from CD or UC patients, who were classified for response (Res) or non-response (Non-res) to the anti-TNFα therapy. Tissues were collected before (Pre-treat) and 4-6 weeks after (Post-treat) their first anti-TNFα treatments. All data are normalized to the condition of healthy tissues (data source: GSE16879). Each datum represents the individual sample collected from the indicated condition. Means ± SEM are shown, and statistical significance is determined by one-way ANOVA test with Dunnett's correction for multiple comparison to the normal healthy tissue. $^{*}P < 0.05$, $^{**}P < 0.01$, $^{***}P < 0.001$.

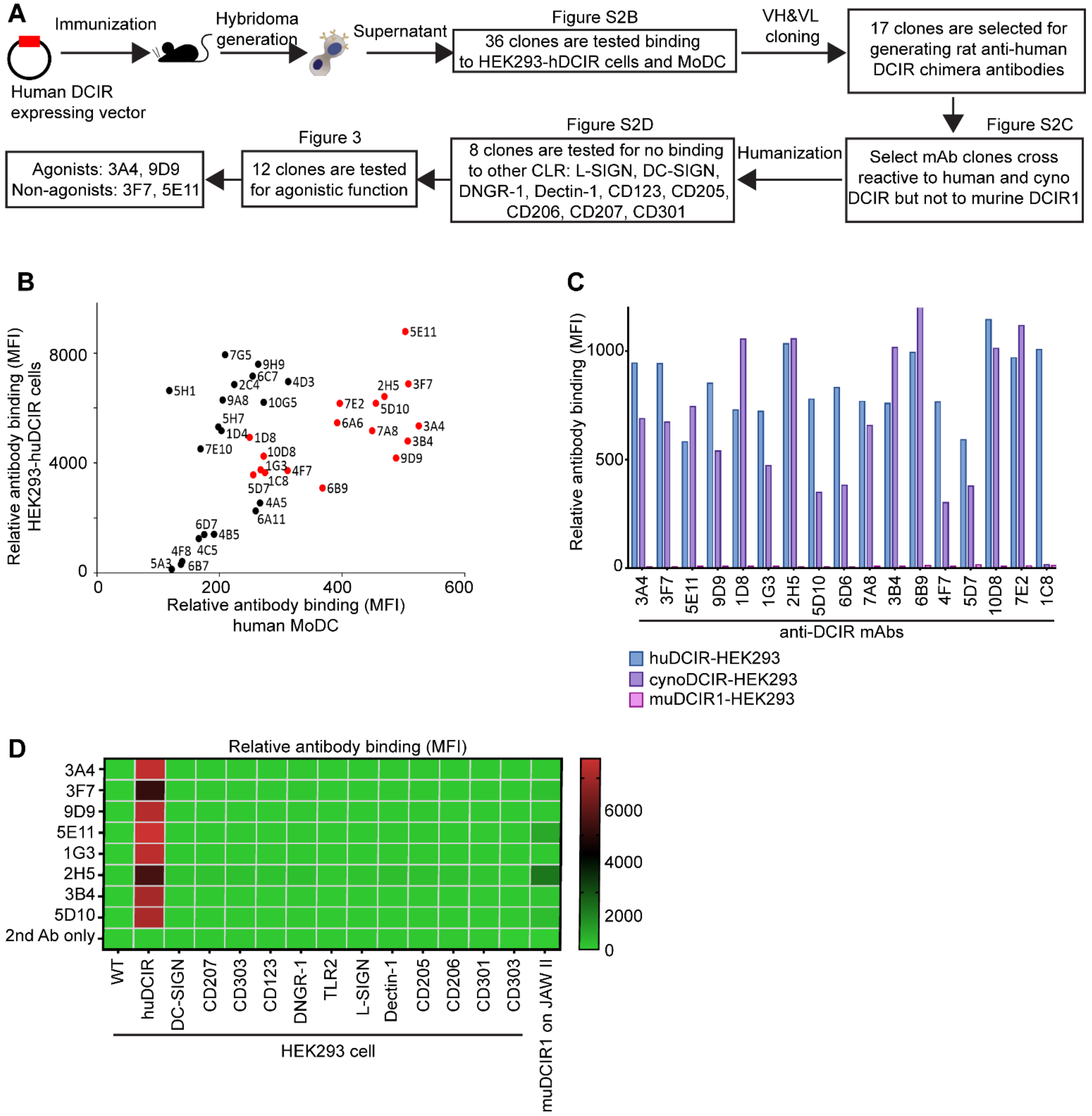

Supplementary Figure 2: Generation and characterization of anti-DCIR mAbs.

(A) Schematic summary of humanized anti-DCIR antibody generation workflow. **(B)** HEK293 cells transfected with huDCIR cDNA vector and human monocyte derived dendritic cells (MoDCs) were incubated with hybridomas' supernatant containing secreted anti-DCIR antibodies for 30min. Binding of the antibodies were quantitated by the fluorescence signal from a A647 anti-rat IgG secondary antibody (10µg/ml) treatment for 30min. Clones secreting strong binder (highlighted in red) were selected for rat anti-huDCIR mAb generation. **(C)** HEK293 cells transfected with *huDCIR*, *cyno DCIR* or *muDcir1* were treated with 10µg/ml rat anti-huDCIR mAbs for 30min. Binding of the antibodies were quantitated by the fluorescence signal from a PE anti-rat IgG secondary antibody (10µg/ml) treatment for 30min. **(D)** HEK293 cells expressing huDCIR or other selected human dendritic cell's receptors, and murine immortal dendritic cell line JAWS II expressing muDCIR1 were

treated with 10µg/ml humanized anti-DCIR mAbs or isotype control for 30min. Binding of the antibodies were quantitated by the fluorescence signal from a A647 anti-human IgG secondary antibody (10µg/ml) treatment for 30min. All data are normalized to the isotype control treated group.

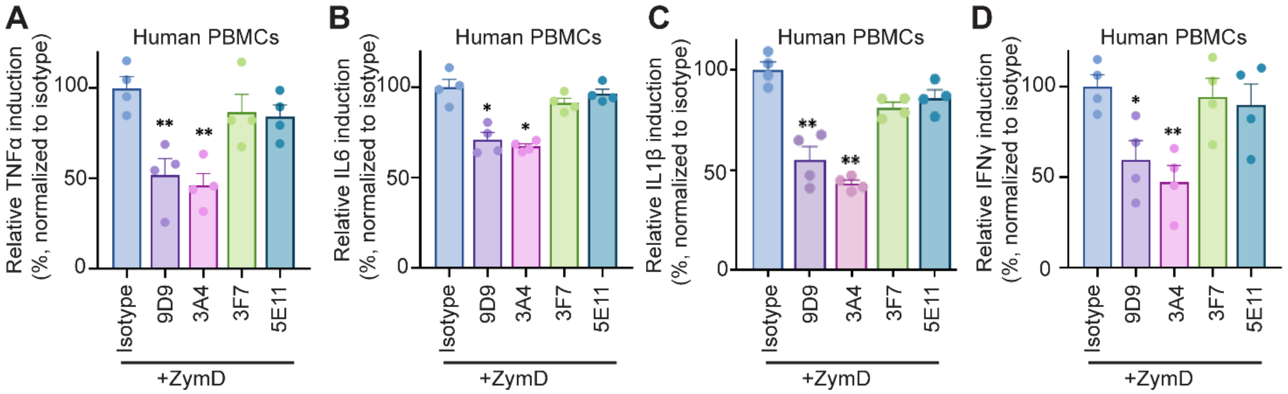

Supplementary Figure 3: Agonistic anti-DCIR mAb provides immunosuppressive function by preventing SHP2 binding to the ITAM.

(A-D) Relative induction of the cytokines secreted from the human PBMC pretreated with 10 μg/ml anti-DCIR mAbs or isotype control for 30 min (n=4/group), followed by 25 μg/ml ZymD stimulation for overnight. All data are normalized to the isotype control treated group. Each datum represents the individual sample. Means ± SEM are shown, and statistical significance is determined by one-way ANOVA test with Dunnett's correction for multiple comparison to the isotype treated condition. *P < 0.05, **P < 0.01, ***P < 0.001.

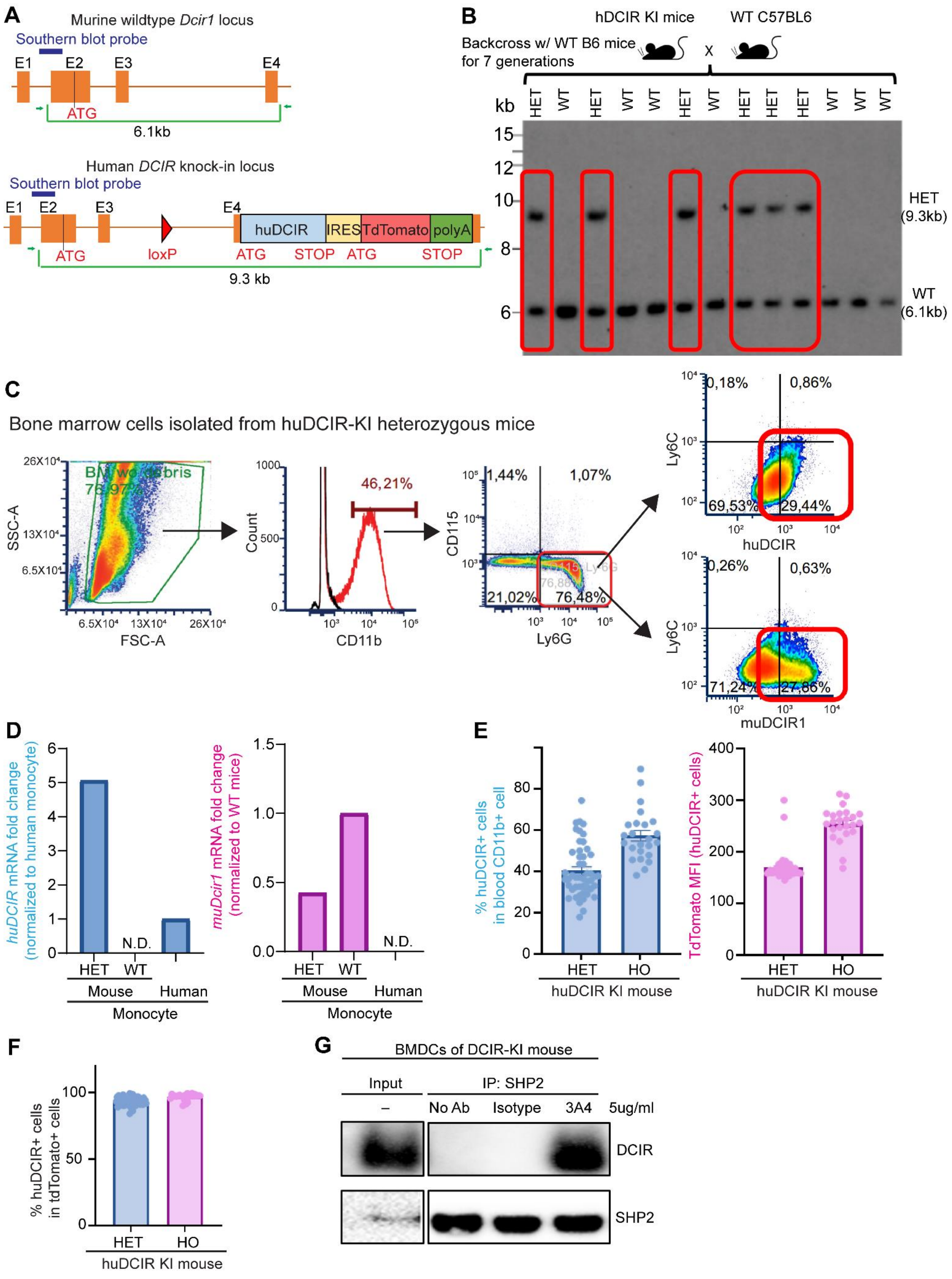

A
Murine wildtype Dcir1 locus
Southern blot probe
E1
E2
E3
E4
ATG
6.1kb
Human DCIR knock-in locus
Southern blot probe
huDCIR
IRES
TdTomato
polyA
loxP
ATG
STOP
9.3 kb
B
hDCIR KI mice
WT C57BL6
Backcross w/ WT B6 mice for 7 generations
x
kb
HET
WT
HET (9.3kb)
WT (6.1kb)
C
Bone marrow cells isolated from huDCIR-KI heterozygous mice
SSC-A
FSC-A
Count
CD11b
46,21%
CD115
Ly6G
1,44%
1,07%
21,02%
76,48%
Ly6C
huDCIR
0,18%
0,86%
69,53%
29,44%
muDCIR1
0,26%
0,63%
71,24%
27,86%
D
huDCIR mRNA fold change (normalized to human monocyte)
N.D.
HET
WT
Mouse
Human
Monocyte
muDcir1 mRNA fold change (normalized to WT mice)
E
% huDCIR+ cells in blood CD11b+ cell
HET
HO
huDCIR KI mouse
TdTomato MFI (huDCIR+ cells)
F
% huDCIR+ cells in tdTomato+ cells
G
BMDCs of DCIR-KI mouse
Input
IP: SHP2
–
No Ab
Isotype
3A4
5ug/ml
DCIR
SHP2

Supplementary Figure 4: Generation and Characterization of huDCIR-KI mice.

(A) Illustrate of the DNA probe designed to detect the *huDCIR* gene in the target locus of *muDCIR1* by Southern blot analysis. **(B)** HET huDCIR-KI male mice were crossbred with WT C57BL/6 female mice for 7 generations before the inbred of HET huDCIR-KI mice to generate HO offspring. Tail clips were analyzed by Southern blot for genotyping. **(C)** Representative flow cytometry of huDCIR and endogenous muDCIR1 expression from bone marrow neutrophils isolated from 2 huDCIR-KI HET mice. **(D)** Relative gene expression of *huDCIR* and *muDcir1* in the murine monocytes from HET huDCIR-KI and WT littermates and human CD14+ monocyte were quantitated by qPCR. Gene expression was normalized to human monocyte (left) or WT mice (right) group. **(E)** Function of the huDCIR-KI locus was confirmed by flow cytometry analysis of huDCIR expression and tdTomato florescence in the blood CD11b+ cells and huDCIR+ cell, respectively, isolated from the HET and HO huDCIR KI mice. **(F)** Flow cytometry analysis of % huDCIR+ cells in tdTomato+ cells isolated from huDCIR HET and HO mice. **(G)** BMDCs from huDCIR-KI mice were treated with 5 µg/ml agonistic anti-huDCIR antibody clone 3A4 and isotype control for 30 min, followed by immunoprecipitation using anti-SHP2 antibody. Interaction between SHP2 and huDCIR was evaluated by the DCIR level identified by the WB. SHP2 from the IP lysate was probed as loading control.

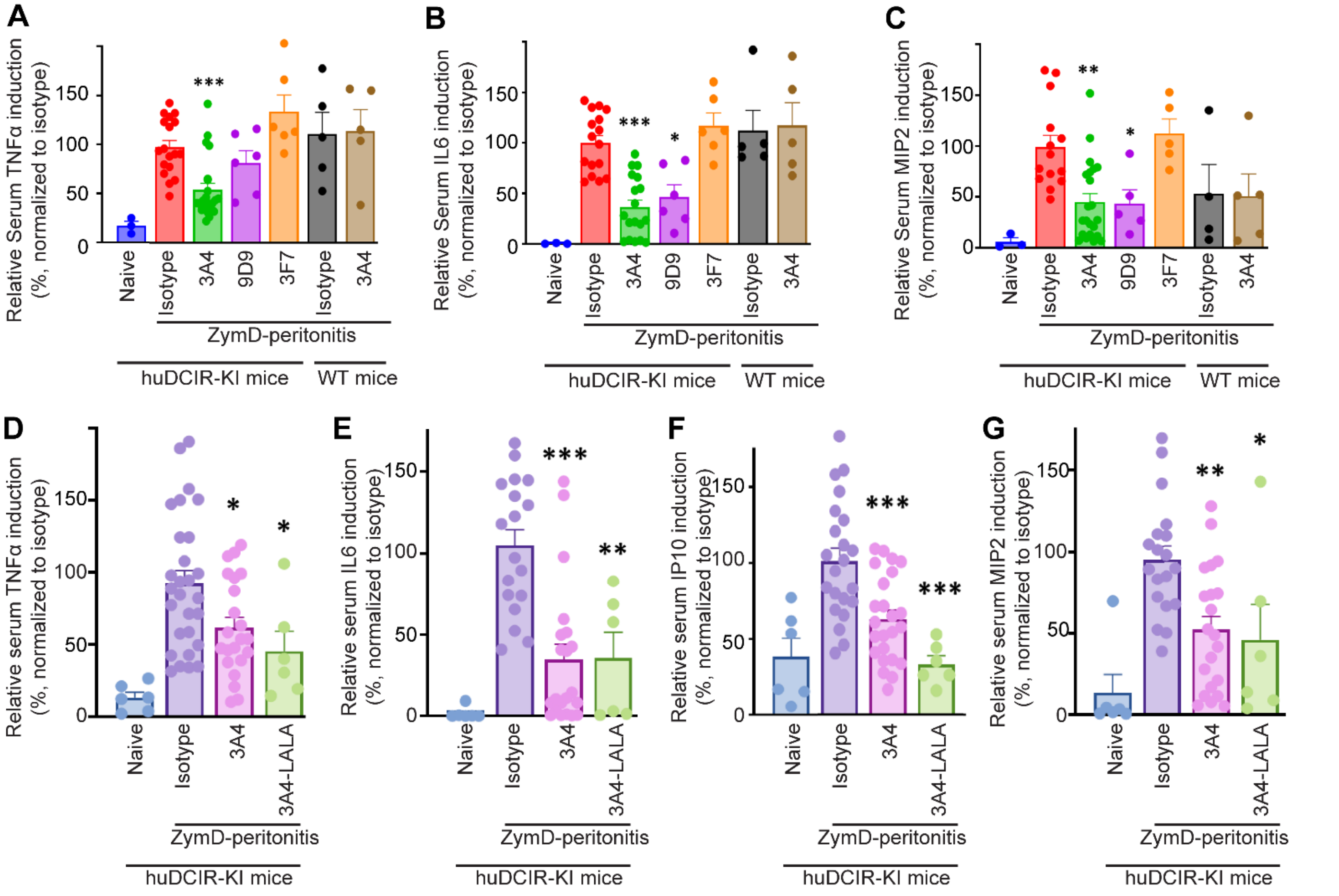

Supplementary Figure 5: Agonistic anti-DCIR mAb ameliorates experimental acute peritonitis.

(A-C) Relative induction of serum cytokines from WT and huDCIR-KI mice received 10mpk (i.p.) anti-DCIR mAbs, 3A4 (n=6 for WT or n=22 for huDCIR-KI), 9D9 (n=6 for huDCIR-KI), 3F7 (n=6 for huDCIR-KI), or isotype control (n=5 for WT or n=18 for huDCIR-KI) in the ZymD-induced peritonitis model. **(D-G)** Relative induction of serum cytokines from huDCIR-KI mice i.p. administrated with 10mpk anti-DCIR mAbs clone 3A4 with WT (n=22) or LALA (L234A and L235A) mutant (n=6) huIgG1 Fc, or isotype (n=20) in the peritonitis model. Data are normalized to the isotype control treated group. Each datum represents the individual mouse treated with indicated condition. Means ± SEM are shown, and statistical significance is determined by one-way ANOVA test with Dunnett's correction for multiple comparison to the isotype treated condition, $*P < 0.05$, $**P < 0.01$, $***P < 0.001$.

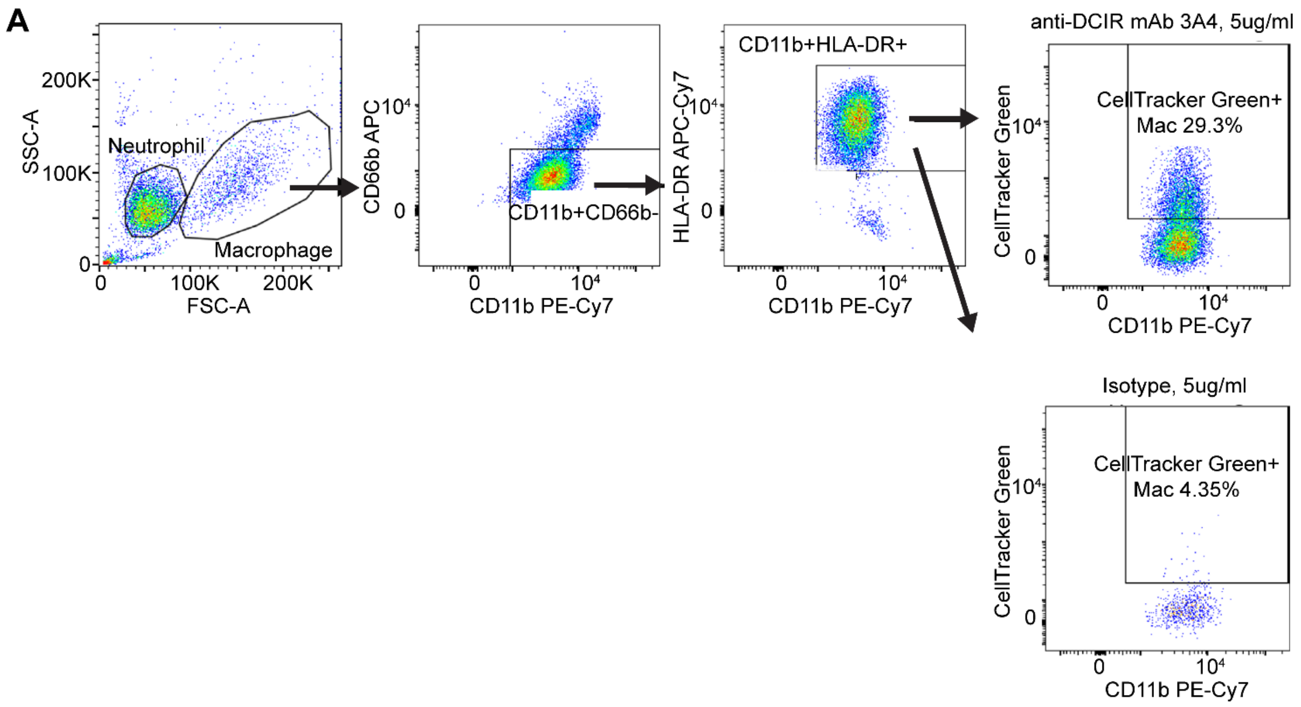

Supplementary Figure 6: Anti-DCIR mAb promotes neutrophils clearance through ADCP.

(A) Representative flow cytometry gating strategy for identifying the macrophage with ADCP effect. CellTracker Green labelled neutrophils were co-cultured with monocyte-derived-macrophage in the presence of 5 µg/ml anti-DCIR mAbs or Fc-matched isotype control for 2 hours.

Supplementary table

Supplementary Table 1: Primers and plasmid sequence

ORF	cDNA Sequence
hDCIR Origene<br>Cat#RC224937<br>NM_016184.4	ATGACTTCGGAAATCACTTATGCTGAAGTGAGGTTCAAAAATGAATTCAAGTCCTCAGGCATCAA<br>CACAGCCTCTTCTGCAGCTTCCAAGGAGAGGACTGCCCCTCTCAAAAGTAATACCGGATTCCCC<br>AAGCTGCTTTGTGCCTCACTGTTGATATTTTTCCTGCTATTGGCAATCTCATTCTTTATTGCTTTT<br>GTCATTTTCTTTCAAAAATATTCTCAGCTTCTTGAAAAAAAGACTACAAAAGAGCTGGTTCATACA<br>ACATTGGAGTGTGTGAAAAAAAATATGCCCGTGGAAGAGACAGCCTGGAGCTGTTGCCCAAAGA<br>ATTGGAAGTCATTTAGTTCCAACTGCTACTTTATTTCTACTGAATCAGCATCTTGGCAAGACAGT<br>GAGAAGGACTGTGCTAGAATGGAGGCTCACCTGCTGGTGATAAACACTCAAGAAGAGCAGGAT<br>TTCATCTTCCAGAATCTGCAAGAAGAATCTGCTTATTTTGTGGGGCTCTCAGATCCAGAAGGTCA<br>GCGACATTGGCAATGGGTTGATCAGACACCATACAATGAAAGTTCCACATTCTGGCATCCACGT<br>GAGCCCAGTGATCCCAATGAGCGCTGCGTTGTGCTAAATTTTCGTAAATCACCCAAAAGATGGG<br>GCTGGAATGATGTTAATTGTCTTGGTCCTCAAAGGTCAGTTTGTGAGATGATGAAGATCCACTTA
Cyno DCIR<br>XM_045364745.1	ATGACTTCAGAAATCACTTATGCTGAAGTGAGGTTCCAAAATGAATCCAAGTTCTCAGGCATCGA<br>CTCTTGGCAATCTCATTCTTTTTTGCTTTTTTCATTTTCTTTCAAAAATATTCTCAGCTTCTTGAAA<br>AAATGACTACGAAAGACCTGGTTCATACAACATTGGAGTGTGTGAAAAAAAATATGACCACGGAA<br>GAGACAGCCTGGAGCTGTTGCCCAAAGAATTGGAAGCCGTTTAGTTCCAACTGCTACTTTATTT<br>CTACTGAATCAGCATCTTGGCAAAAGAGTGAGAAGGACTGCGCTAGAATGGAGGCTCACCTGCT<br>GGTGATAAACACTCGAGAAGAGCAGGATTTCATCTTCCAGAATCTGCAAGAAGAATCTGCTTATT<br>TTGTGGGGCTCTCAGATCCAGAAGGTCAGCGACATTGGCAATGGGTAGATCGGACACCATACA<br>ATGAAAGTTCCACATTCTGGCATCCACATGAGCCCAGTGATCCCGATGAGCGCTGTGTTGTGCT<br>AAATTTCCGTAAAACACCTAAAAGATGGGGCTGGAATGATGTTCATTGTATTGTTCATCAAAGGT<br>CAGTTTGTGAGATGATGAAGATCCACTTATGA
hDectin1 Origene<br>Cat#SC307610:<br>NM197947	ATGGAATATCATCCTGATTTAGAAAATTTGGATGAAGATGGATATACTCAATTACACTTCGACTCT<br>CAAAGCAATACCAGGATAGCTGTTGTTTCAGAGAAAGGATCGTGTGCTGCATCTCCTCCTTGGC<br>GCCTCATTGCTGTAATTTTGGGAATCCTATGCTTGGTAATACTGGTGATAGCTGTGGTCCTGGGT<br>ACCATGGCTATTTGGAGATCCAATTCAGGAAGCAACACATTGGAGAATGGCTACTTTCTATCAAG<br>AAATAAAGAGAACCACAGTCAACCCACACAATCATCTTTAGAAGACAGTGTGACTCCTACCAAAG<br>CTGTCAAAACCACAGGGGTTCTTTCCAGCCCTTGTCCTCCTAATTGGATTATATATGAGAAGAGC<br>TGTTATCTATTCAGCATGTCACTAAATTCCTGGGATGGAAGTAAAAGACAATGCTGGCAACTGGG<br>CTCTAATCTCCTAAAGATAGACAGCTCAAATGAATTGGGATTTATAGTAAAACAAGTGTCTTCCC<br>AACCTGATAATTCATTTTGGATAGGCCTTTCTCGGCCCCAGACTGAGGTACCATGGCTCTGGGA<br>GGATGGATCAACATTCTCTTCTAACTTATTTCAGATCAGAACCACAGCTACCCAAGAAAACCCAT<br>CTCCAAATTGTGTATGGATTCACGTGTCAGTCATTTATGACCAACTGTGTAGTGTGCCCTCATAT<br>AGTATTTGTGAGAAGAAGTTTTCAATGTAA
M musculus Dcir1<br>(Clec4a2) Origene<br>Cat#MC200710<br>NM_001170333.2	ATGGCAAGCGAGATCACCTACGCCGAAGTGAAGTTCAAAAACGAGAGCAACTCCCTTCATACCT<br>ATAGCGAGTCACCAGCTGCCCCTAGGGAGAAGCCTATCCGCGATTTGCGCAAGCCGGGCTCCC<br>CTTCCCTGCTCCTTACGTCTCTGATGCTGCTCCTGCTCTTGCTGGCTATCACCTTCCTGGTTGCC<br>TTCATCATTTATTTCCAGAAGTATTCTCAGCTCCTGGAAGAGAAAAAGGCCGCTAAGAACATCAT<br>GCACAACGAGTTGAACTGTACTAAGTCAGTCAGTCCCATGGAAGCACCCCCTATCGGGCAAAG<br>GGCCCTGACCCTGGAATCTATCGAGATTGACTTGGGTATCCTGGCTCCCGAGGACAAAGTCTG<br>GAGCTGTTGCCCGAAGGATTGGCGCCTTTTTGGGTCTCATTGTTACCTCGTGCCTACAGTCAGC<br>TCTTCCGCTTCCTGGAACAAGTCAGAAGAGAACTGCTCCCGGATGGGGGCTCACCTCGTGGTT<br>ATCCAGTCTCAAGAAGAGCAAGACTTTATTACCGGCATCTTGGATACCCACGCCGCTTATTTCAT<br>CGGACTGTGGGATACCGGCCACCGTCAATGGCAGTGGGTGGACCAAACCCCCTACGAAGAGA<br>GCATCACATTTTGGCATAACGGTGAGCCGAGCAGTGGGAACGAGAAGTGCGCCACCATTATCT<br>ATAGGTGGAAGACCGGCTGGGGCTGGAACGACATCTCCTGCAGCCTGAAGCAGAAATCCGTGT<br>GTCAGATGAAAAAGATCAACCTG
ITIM Sequences from DCIR	ATGACCAGCGAAATTACCTATGCGGAAGTGCGCTTTAAAAACGAATTTAAAAGCAGCGGCATTA<br>ACACCGCGAGCAGCGCGGCGAGCAAAGAACGCACCGCGCCGCATAAAAGCAACACC
ITAM sequences from DECTIN1	ATGGAATATCATCCGGATCTGGAAAACCTGGATGAAGATGGCTATACCCAGCTGCATTTTGATA<br>GCCAGAGCAACACCCGCATTGCGGTGGTGAGCGAAAAAGGCAGCTGCGCGGCGAGC

Primers	Forward	Reverse
Cloning Dectin-1 ITAM	AATTCATGGAGTATCATCCCGATTTAGAGAATTTAGACGAGGATGGCTACA<br>CCCAGCTGCACTTCGACAGCCAGAGCAACACCAGAATCGCCGTCGTGAG<br>CGAGAAAGGCTCTTGTGCCGCTAGCGGCTTCCCT	GCGGCCGCTCA<br>GAGGTGGATCT<br>TCATC

Supplementary Table 2: Confocal laser endomicroscopy (CLE) scoring criteria

Status	Score
100% crypt loss; 100% NE680$^+$ cells with erosion	5
Significant presence of collapsed crypts; high NE680 signal spreading in erosion area; >50% of crypts lack acriflavine staining	4
Significant presence of collapsed crypts; high NE680 signal in crypt lumen and spreading in erosion area	3
Presence of collapsed crypts; high NE680 signal in crypt lumen	2
Healthy crypt structure; low NE680 signal in crypt lumen	1
Well intact crypt architecture & absence of NE680 signal	0

Supplementary files

Supplementary Movie 1: Crypt infiltrated DCIR+ cells in naïve mice.

Related to Figure 4H and I. AF488 anti-huDCIR mAb (1G3) was i.v. injected into huDCIR-KI mice on Day 3. Representative movie taken by the CLE shows no AF488 anti-DCIR mAb (green) bound cells at the steady state (Day 0 before DSS). Green: AF488 anti-DCIR mAb (huDCIR+ cell). Red: AF680 dextran (blood vessel).

Supplementary Movie 2: Crypt infiltrated DCIR+ cells during the DSS-induced colitis.

Related to Figure 4H and I. AF488 anti-huDCIR mAb (1G3) was i.v. injected into huDCIR-KI mice on Day 3 and 6 during the DSS-induced colitis. Representative movie taken by the CLE shows AF488 anti-DCIR mAb (green) bound cells during the colitis (Day-7 post DSS). Green: AF488 anti-DCIR mAb (huDCIR+ cell). Red: AF680 dextran (blood vessel).

Supplementary Movie 3: Agonistic anti-DCIR mAb attenuates experimental colitis. (Naïve)

Related to Figure 7D. Representative movie taken by CLE examining the integrity of colon crypts (green, labeled by acriflavine) and accumulation of neutrophils (red, labeled by NE680) from the mice with no DSS colitis.

Supplementary Movie 4: Agonistic anti-DCIR mAb attenuates experimental colitis. (DSS, Isotype)

Related to Figure 7D. Representative movie taken by CLE examining the integrity of colon crypts (green, labeled by acriflavine) and accumulation of neutrophils (red, labeled by NE680) from the mice treated with isotype control during the colitis (Day 7 post DSS).

Supplementary Movie 5: Agonistic anti-DCIR mAb attenuates experimental colitis. (DSS, 9D9)

Related to Figure 7D. Representative movie taken by CLE examining the integrity of colon crypts (green, labeled by acriflavine) and accumulation of neutrophils (red, labeled by NE680) from the mice treated with agonistic anti-DCIR mAb (9D9) during the colitis (Day 7 post DSS).

Supplementary Movie 6: Agonistic anti-DCIR mAb attenuates experimental colitis. (DSS, 3F7)

Related to Figure 7D. Representative movie taken by CLE examining the integrity of colon crypts (green, labeled by acriflavine) and accumulation of neutrophils (red, labeled by NE680) from the mice treated with non-agonistic anti-DCIR mAb (3F7) during the colitis (Day 7 post DSS).
