# Supplementary material for: Agonistic anti-DCIR antibody inhibits ITAM-mediated inflammatory signaling and promotes immune resolution
Source: JCI Insight. 2024 May 23;9(12):e176064. doi: 10.1172/jci.insight.176064 (PMC11383175; doi:10.1172/jci.insight.176064)

Westernblot for Figure 2B

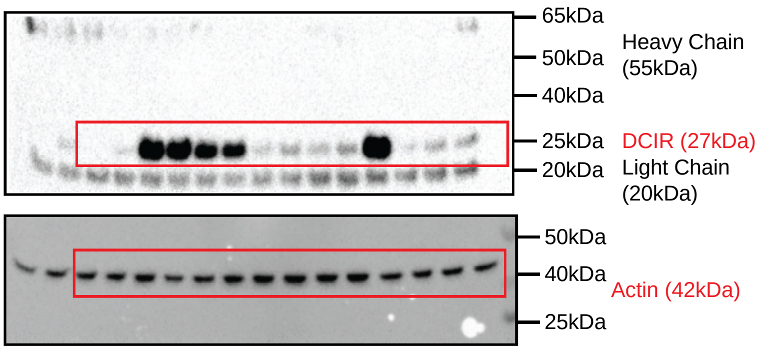

Westernblot for Figure 2C

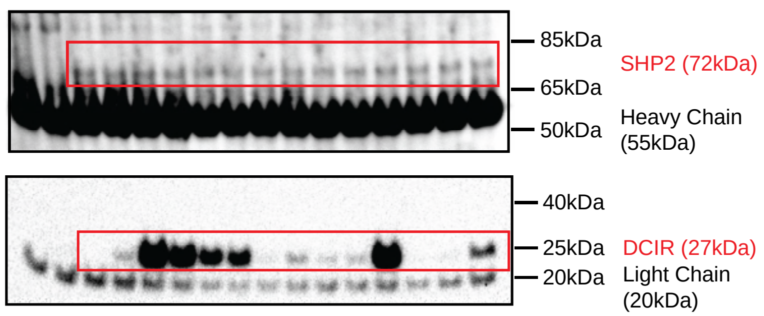

Westernblot for Figure 3C

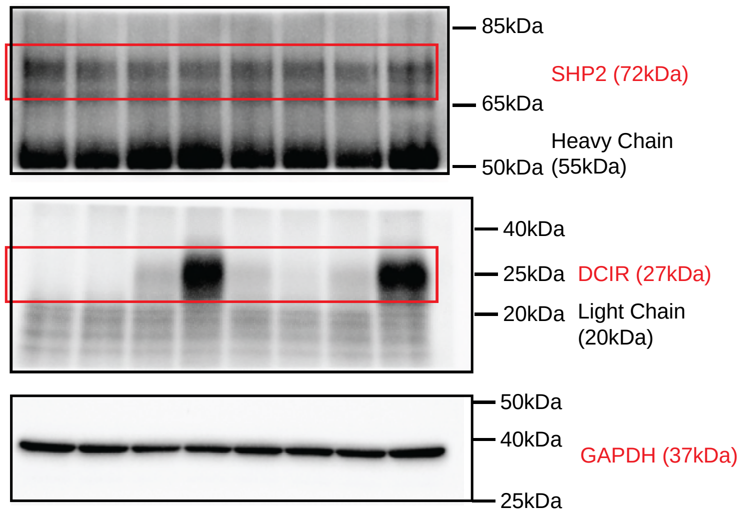

Westernblot for Figure 3D

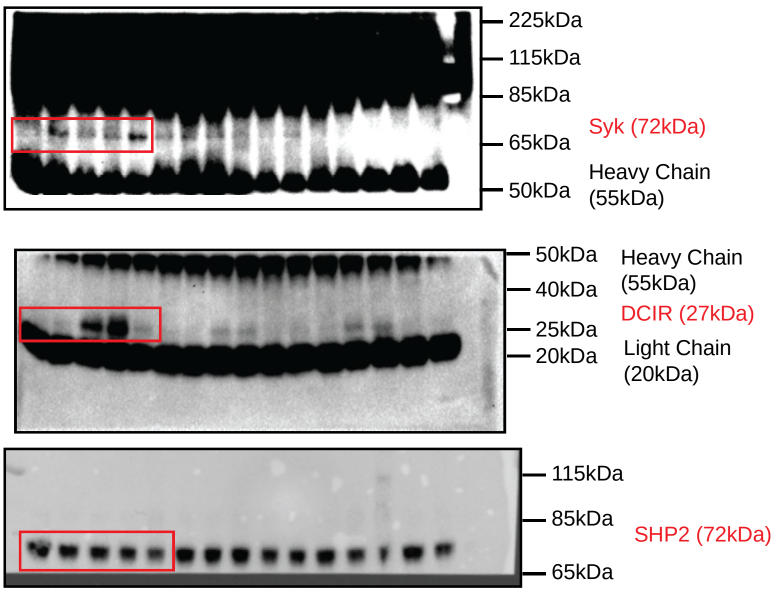

Westernblot for Figure 3E

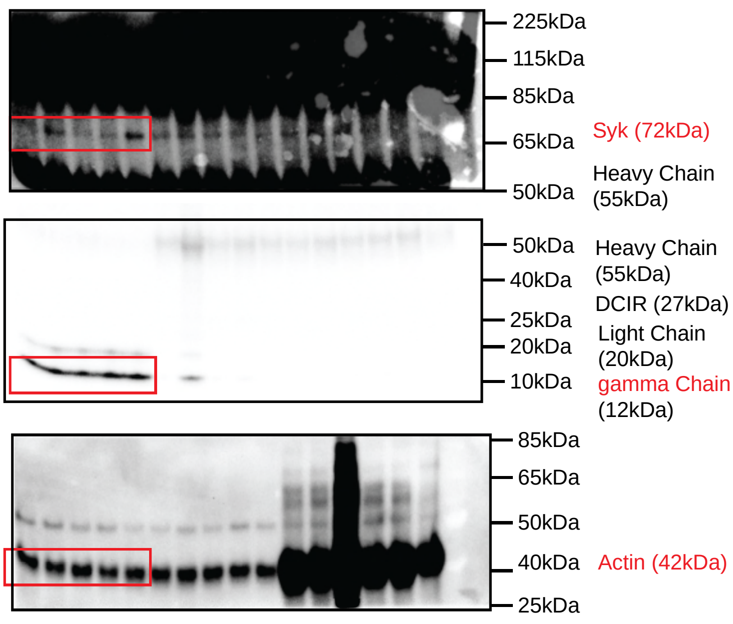

Westernblot for Supplementary Figure 4E

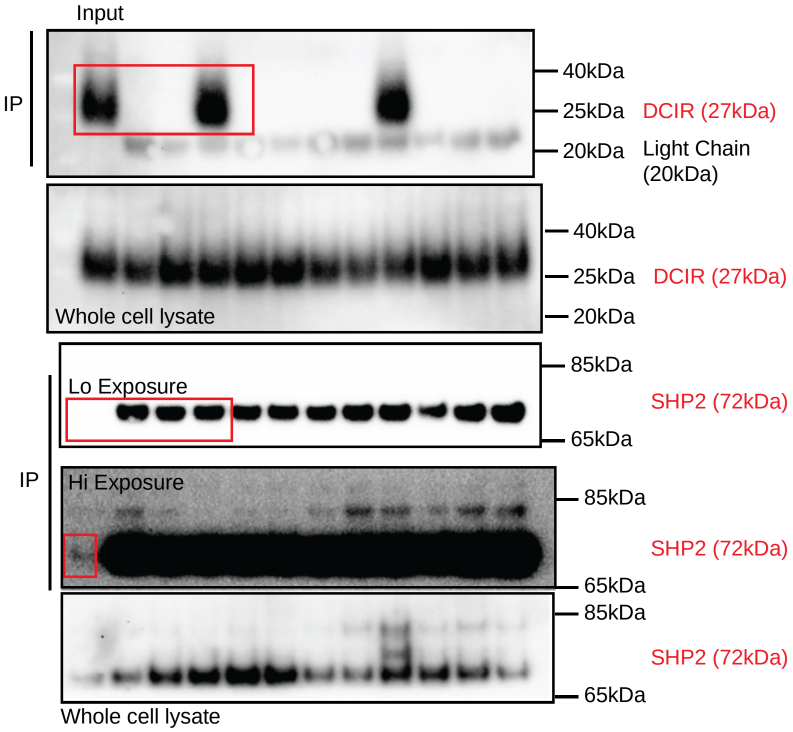

Supplement: Unedited blot and gel images [file jciinsight-9-176064-s072.pdf]
